# *HMCN1* variants aggravate epidermolysis bullosa simplex phenotype

**DOI:** 10.1084/jem.20240827

**Published:** 2025-02-20

**Authors:** Shir Bergson, Ofer Sarig, Moshe Giladi, Janan Mohamad, Mariana Mogezel-Salem, Karina Smorodinsky-Atias, Ofir Sade, Bar Manori, Sari Assaf, Kiril Malovitski, Yarden Feller, Mor Pavlovsky, Stefan Hainzl, Thomas Kocher, Julia I. Hummel, Noy Eretz Kdosha, Lubna Gazi Khair, Roland Zauner, Josefina Pinon Hofbauer, Ruby Shalom-Feuerstein, Verena Wally, Ulrich Koller, Liat Samuelov, Yoni Haitin, Uri Ashery, Rotem Rubinstein, Eli Sprecher

**Affiliations:** 1 Division of Dermatology, Tel Aviv Medical Center, Tel Aviv, Israel; 2 Faculty of Medical and Health Sciences, Tel Aviv University, Tel Aviv, Israel; 3 Department of Internal Medicine D, Tel Aviv Medical Center, Tel Aviv, Israel; 4 Faculty of Life Sciences, School of Neurobiology, Biochemistry and Biophysics, Tel Aviv University, Tel Aviv, Israel; 5 Department of Dermatology and Allergology, EB House Austria, Research Program for Molecular Therapy of Genodermatoses, University Hospital of the Paracelsus Medical University Salzburg, Salzburg, Austria; 6 The Rappaport Faculty of Medicine, Technion, Israel Institute of Technology, Haifa, Israel; 7 Sagol School of Neuroscience, Tel Aviv University, Tel Aviv, Israel

## Abstract

Epidermolysis bullosa simplex (EBS) refers to a heterogeneous group of inherited skin disorders characterized by blister formation within the basal cell layer. The disease is characterized by marked variations in phenotype severity, suggesting co-inheritance of genetic modifiers. We identified three deleterious variants in *HMCN1* that co-segregated with a more severe phenotype in a group of 20 individuals with EBS caused by mutations in *KRT14*, encoding keratin 14 (K14). *HMCN1* codes for hemicentin-1. Protein modeling, molecular dynamics simulations, and functional experiments showed that all three *HMCN1* variants disrupt protein stability. Hemicentin-1 was found to be expressed in human skin above the BMZ. Using yeast-2-hybrid, co-immunoprecipitation, and proximity ligation assays, we found that hemicentin-1 binds K14. Three-dimensional skin equivalents grown from hemicentin-1–deficient cells were found to spontaneously develop subepidermal blisters, and *HMCN1* downregulation was found to reduce keratin intermediate filament formation. In conclusion, hemicentin-1 binds K14 and contributes to BMZ stability, which explains the fact that deleterious *HMCN1* variants co-segregate with a more severe phenotype in *KRT14*-associated EBS.

## Teaser

Mutations in the *KRT14* gene cause epidermolysis bullosa simplex (EBS), featuring abnormal basement membrane zone (BMZ) function. We identified deleterious variants in *HMCN1* co-segregating with severe EBS and found that HMCN1 directly binds keratin 14 and critically contributes to the BMZ integrity.

## Introduction

Epidermolysis bullosa (EB) refers to a large clinically and genetically heterogeneous group of hereditary skin blistering conditions (Bardhan et al., 2020; Harvey et al., 2022). Blisters occur secondary to exposure of the skin to mechanical trauma and heat. EB is traditionally classified into clinicopathological subgroups based on the location of the blisters (Has et al., 2020). EB simplex (EBS) is associated with blister formation at the level of the basal cell layer, junctional EB results from epidermal–dermal separation at the level of the lamina lucida, and dystrophic EB is due to blister formation within the upper dermis (Has et al., 2020).

EBS is the most common form of EB (Sprecher, 2010). Mild forms of EBS feature blisters involving primarily the soles, while more severe cases are associated with widespread blistering of the skin and even mucosal tissue (Has et al., 2020; Sprecher, 2010). Most cases of EBS are due to pathogenic variants in *KRT5* (Lane et al., 1992) and *KRT14* (Bonifas et al., 1991; Coulombe et al., 1991, 2009), which encode basal cell keratin intermediate filaments, although additional genes have been associated with EBS pathogenesis as well (Bardhan et al., 2020).

Clinical variability in EBS (Coulombe et al., 2009) has been attributed to the nature and location of the causative variant (Chen et al., 2023; Sørensen et al., 1999). However, individuals, including siblings, carrying the same EBS-causing variant may still display variable degrees of severity. Occupation, climate, and other environmental factors may account in part for clinical variability in these cases (Bardhan et al., 2020). Moreover, genetic and epigenetic factors may also modify the phenotypic expression of causative variants in inherited diseases, including various forms of EB (Bochner et al., 2017; Floeth and Bruckner-Tuderman, 1999; Kousi and Katsanis, 2015; Magrinelli et al., 2021; Sproule et al., 2023a, 2023b; Trerotola et al., 2015; Zekry et al., 2023). A polymorphism in the *MMP1* gene encoding collagenase has been found to aggravate recessive dystrophic EB (RDEB) phenotype (Kern et al., 2009; Titeux et al., 2008). High TGF-β activity may also contribute to RDEB severity (Cianfarani et al., 2019; Odorisio et al., 2014). Additionally, many reports have demonstrated that digenic inheritance of pathogenic variants in EBS-associated genes could result in intrafamilial clinical variability (Kim et al., 2017; Padalon-Brauch et al., 2012; Yu et al., 2020).

Clearly, the identification of genetic modifiers is not only of importance to the genetic counseling of families at risk for inherited conditions, but it may also uncover potentially actionable pathways (McCabe, 2017). Here, we identified deleterious variants in *HMCN1*, encoding hemicentin-1, as genetic modifiers of *KRT14*-associated EBS.

## Results

### HMCN1 variants co-segregate with disease severity in EBS

We ascertained a total of 90 EB families including 195 patients. All participants had been exome sequenced and, in all cases, a causative variant in one EB-associated gene had been detected (Bergson et al., 2023). We identified 107 cases sharing a causative variant with at least one other patient in the cohort. Among these, seven families in which two or more individuals shared the same variant but displayed a divergent phenotype were identified and screened for pathogenic variants found in any gene not associated with EB and co-segregating with the disease severity phenotype. Using this strategy, we identified three variants in *HMCN1* that co-segregated with the disease phenotype severity in four families.

**Figure 1.**
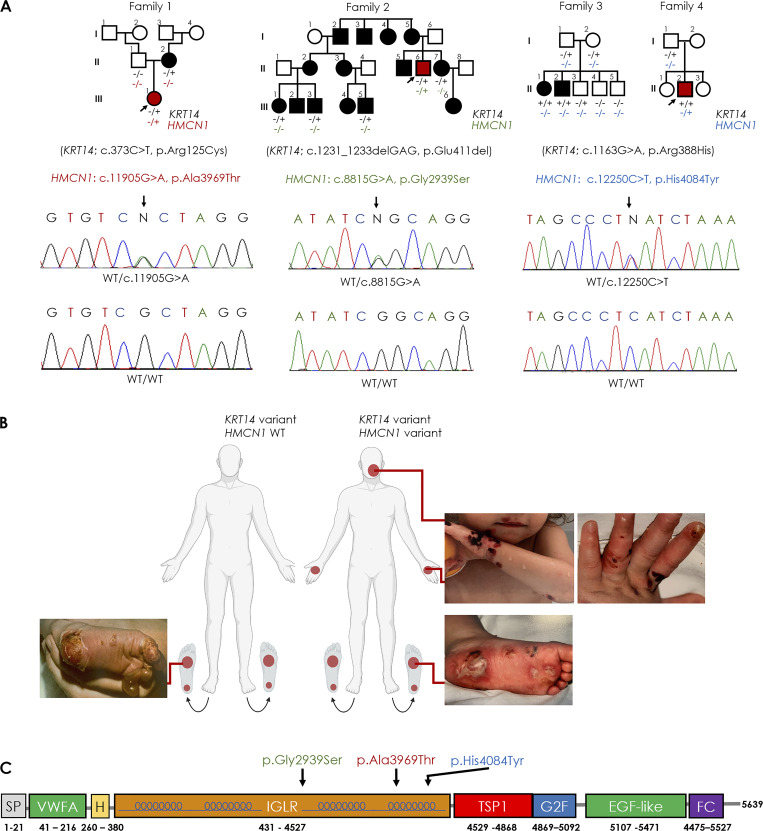
**Pedigrees, clinical features, and variant analysis. (A)** Pedigrees of the four families. Black symbols denote affected individuals, red symbols denote more severely affected individuals. Arrows point to probands in each family. Genotypes are indicated for those individuals who consented to participate in the study. Below are electropherograms obtained through direct sequencing of *HMCN1* that revealed a heterozygous G>A transition (arrow) at position c.11905 of the cDNA sequence in individual III–1, family 1, a heterozygous G>A transition (arrow) at position c.8815 of the cDNA sequence in individual II-6, family 2, as well as a heterozygous C>T transition (arrow) at position c.12250 of the cDNA sequence in individual II-2, family 4 (middle panels). The WT sequences (WT/WT) are given for comparison (lower panels). **(B)** Mildly affected individuals featured plantar involvement only while more severe cases carry variants in *HMCN1* and showed at the same age, severe involvement of the hands and additional skin areas. **(C)** The predicted amino acid changes and the location of the three variants are depicted along a schematic representation of hemicentin-1 and its domains (SP, signal peptide; H, hemicentin domain; IGLR, tandem Ig-like repeat; TSP1, thrombospondin type1 repeat domain; G2F, G2 fragment domain; FC, fibulin carboxy-terminal domains; modified from Xu et al. [2013]).

Those four families included 20 individuals affected with EBS due to pathogenic variants in *KRT14* (heterozygous c.373C>T, p.Arg125Cys in family 1; heterozygous c.1231_1233delGAG, p.Glu411del in family 2; and homozygous c.1163G>A, p.Arg388His in family 3 and 4) (Fig. 1 A). Haplotype analysis indicated that c.1163G>A, p.Arg388His originates from a common ancestor in families 3 and 4 (founder effect as previously described [Ciubotaru et al., 2003]) (Table S1). All affected individuals displayed blistering over the plantar skin surface. In addition, three individuals also displayed widespread skin involvement over the years and at the same age compared with less severely affected individuals (Family 1, III-1; Family 2, II-6, Family 4, II-2) (Fig. 1 B). The more severely affected individuals displayed in addition to plantar skin involvement, persistent blistering over the palms and fingers, occasionally associated with involvement of the face and toe dystrophy (Fig. 1 B). These three individuals were shown to carry the following heterozygous variants in *HMCN1*: c.11905G>A, p.Ala3969Thr (Family 1, III-1); c.8815G>A, p.Gly2939Ser (Family 2, II-6); c.12250C>T, p.His4084Tyr (Family 4, II-2) (Fig. 1 A).

*HMCN1* encodes hemicentin-1, a member of the fibulin family of large extracellular matrix proteins. Hemicentins share a number of structural motifs (Fig. 1 C): an N-terminal highly conserved von Willebrand factor A (VWFA) domain, which is considered to mediate binding of hemicentin partners (Dong et al., 2006); a hemicentin motif and a long stretch (>40) of tandem immunoglobulin (Ig) domains, which regulates the rigidity of the protein and affects its ability to bind other proteins; a G2F domain associated with multiple tandem epidermal growth factor (EGF) domains, implicated in protein polymerization and track formation; and a fibulin C-terminal module domain (Dong et al., 2006; Lin et al., 2020; Whittaker and Hynes, 2002; Xu and Vogel, 2011; Xu et al., 2013). Functional studies carried out in *Caenorhabditis elegans*, zebrafish, and mouse have assigned hemicentin-1 an essential role in basement membrane formation and integrity (Gianakas et al., 2023; Vogel and Hedgecock, 2001; Welcker et al., 2021; Zhang et al., 2022). Hemicentin has been shown in nematodes to organize hemidesmosomes formation in different tissues and to interact with various components of the basement membrane (Gianakas et al., 2023).

Of note, polymorphisms in the *HMCN1* gene, localized to the EGF domains, have been found to be associated with a higher propensity to develop age-related macular degeneration (Iyengar et al., 2004; Schultz et al., 2003). The patients (aged 12–40 years) found in this study to carry *HMCN1* variants did not show any signs of macular injury upon fundus examination (not shown). Of note, the *HMCN1* variants associated with age-related macular degeneration are located in regions distinct from those harboring the EBS-associated variants (Iyengar et al., 2004; Schultz et al., 2003).

### HMCN1 variants affect hemicentin-1 structure or expression

The three *HMCN1* variants co-segregating with EBS severity were found to localize to the tandem Ig domains (Fig. 1 C). All three variants are rare, and based on several prediction software (including GERP++, PROVEAN, SIFT4G, PolyPhen2, and CADD), they are likely to exert a deleterious effect on protein function (see detailed predictions in Table S2).

Of note, we screened an in-house dataset of exomes obtained in a population of 8,011 healthy individuals (who had undergone a face-to-face interview before exome sequencing). We identified among those individuals, 185 individuals who were found to be carriers of one of the three *HMCN1* variants. None of these individuals reported any abnormal cutaneous or non-cutaneous sign (Table S2), which indicates that deleterious variants in *HMCN1* are not in themselves sufficient to cause skin blistering.

**Figure 2.**
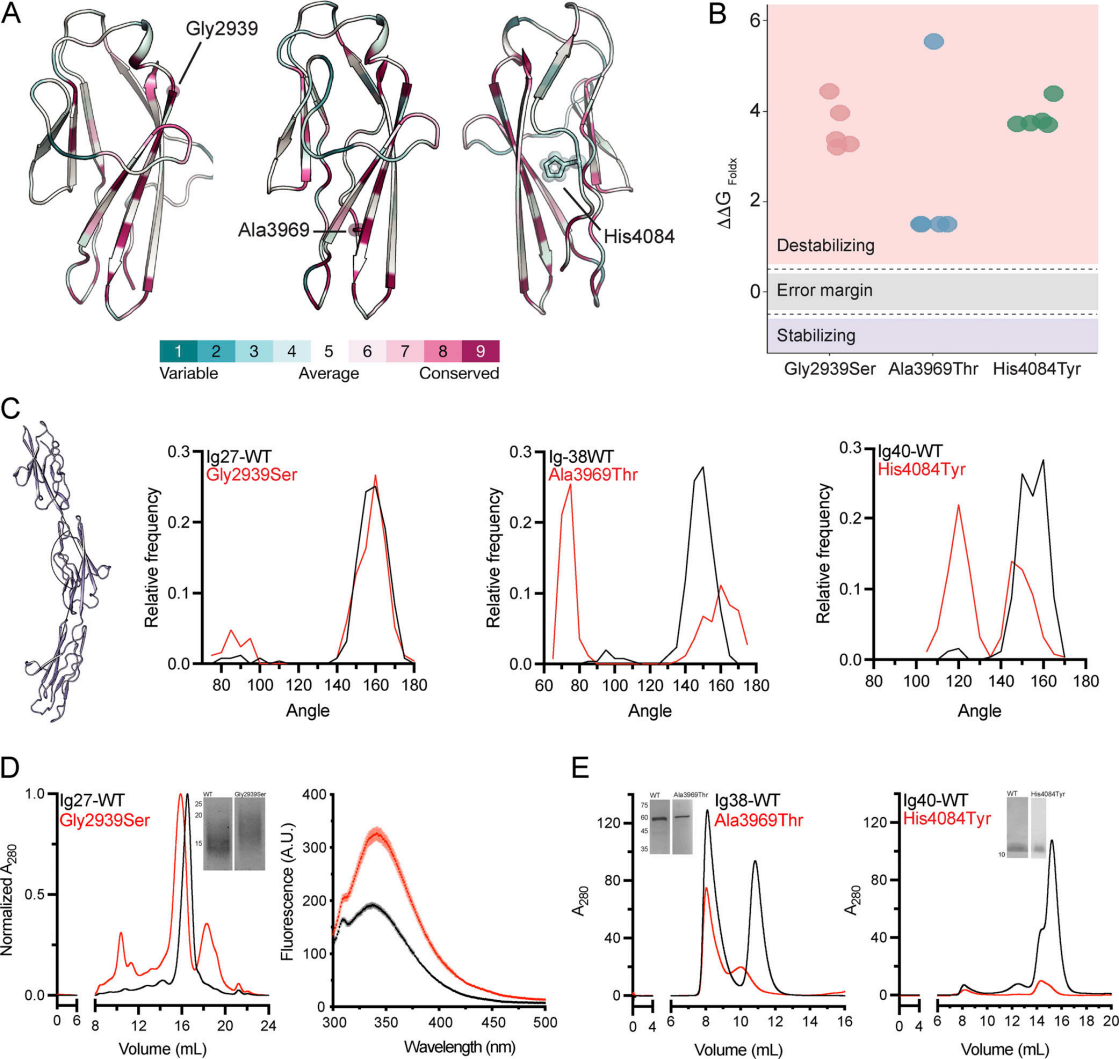
**Protein modeling, dynamics simulation, and functional studies. (A)** Evolutionary conservation analysis mapped onto the predicted protein structures. **(B)** The effect of *HMCN1* variants on protein stability was calculated by FoldX (ΔΔG). All three variants are predicted to destabilize the protein substantially. **(C)** A scheme of an Ig tandem used for MD simulations is presented (left panel). Each of the Ig domains bearing a variant along with the two flanking Ig domains was modeled (with and without the deleterious variant) and used to run 250-ns MD simulations while measuring the interdomain angle as indicated in the left panel. The frequency distribution of the interdomain angle along the simulation trajectories is shown. Black curves denote the WT proteins, while the red curves denote the mutants as indicated. Note that the amino acid substitution causes significant deviations from the straight axis of the constructs harboring the Ig38 and Ig40 Ig domains compared to WT. **(D)** Structural studies of hemicentin-1 Ig27 domain are presented (each experiment was repeated three times). The left panel shows the elution profiles from size-exclusion chromatography for Ig27 WT (black) and mutant (red) domains. Inset, SDS-PAGE of purified Ig27-WT and Ig27-Gly2939Ser; molecular masses in kDa are indicated to the left. Note that the mutant domain shows an apparent larger molecular weight compared to WT. The right panel shows tryptophan fluorescence analysis of Ig27 WT (black) and mutant (red) domains, revealing a significant shift in emission wavelength and amplitude of the mutant domain as compared with the WT domain. **(E) **Purified Ig38 and Ig40 WT and mutants (p.Ala3969Thr and p.His4084Tyr, respectively) domains were analyzed by size exclusion chromatography (each experiment was repeated three times). Inset, SDS-PAGE analysis; molecular masses in kDa are indicated to the left. Note the reduced amount of Ig38 and absent Ig40 protein, as corroborated by the nickel exclusion chromatography data (Fig. S1). Source data are available for this figure: SourceData F2.

Previous data on proteins featuring tandem Ig domains indicate that these repeats are rigidly oriented. This structural property is crucial for the function of such proteins and their ability to bind to other proteins. The Ig tandem of titin is the best-studied example (Brunello and Fusi, 2023), where Ig domains are rigidly connected along a straight axis, which in turn is essential for the mechanical properties of the protein (von Castelmur et al., 2008). To test if the tandem Ig region of hemicentin-1 exhibits a similar structural organization and how this may be perturbed by the identified variants, we first used alphafold2 (Jumper et al., 2021) to generate structural models. These models focused on each Ig domain bearing a variant individually, as well as in combination with the two flanking Ig domains so to capture local and interdomain effects. Protein modeling revealed that p.Gly2939 and p.Ala3969 are in structural proximity to the adjacent Ig domain interface while the third variant, p.His4084, is positioned at the Ig domain core (Fig. 2 A). In addition, we found that all three *HMCN1* variants positions are conserved (Fig. 2 A). We then employed FoldX (Schymkowitz et al., 2005) to predict the impact of each variant on protein stability. The predictions indicated significant destabilizing effects for all three variants, with Δ ΔG_bind_ of ∼1.5 kcal/mol for p.Ala3969 and exceeding 3 kcal/mol for p.Gly2939 and p.His4084 (Fig. 2 B). To further test the impact of the three variants on structural stability, we performed molecular dynamics (MD) simulations (Fig. 2 C). We measured the angle between cysteine residues forming a disulfide bridge at the center of each domain along the 250-ns simulations trajectories (Fig. 2 C). Importantly, all modeled WT Ig domain tandems exhibit a rigid and elongated conformation with an interdomain angle of ∼150° along the simulation trajectory (Fig. 2 C). While p.Gly2939Ser shows a similar domain orientation as compared to the WT along the simulation, the p.Ala3969Thr and p.His4084Tyr variants result in frequent bending of the domains, resulting in a bimodal distribution of the protein conformation, indicative of reduced rigidity and significantly disturbed mechanical stability of the protein (Fig. 2 C).

As the p.Gly2939Ser did not exhibit altered conformational distribution according to the MD simulations, we sought to further analyze the structural effect of this mutation at the Ig27 domain level. Therefore, we purified both WT and p.Gly2939Ser variant-harboring Ig27 in mammalian cells (Fig. 2 D). Strikingly, SDS-PAGE and size-exclusion chromatography analyses revealed that the variant exhibits a substantial mass shift, with a significantly slower migration pattern and earlier elution volume (Fig. 2 D, left panel). Notably, this effect may be associated with a novel O-glycosylation site introduced by the variant. Finally, we employed tryptophan fluorescence spectroscopy analysis, which ascertains the effect of the local chemical environment and is thus a sensitive approach to probe the conformational state of proteins (Vivian and Callis, 2001). Notably, the Ig27 construct used here contains one tryptophan residue, W2906, adjacent to p.Gly2939Ser. Intriguingly, the p.Gly2939Ser variant results in maximal fluorescence peak shift and variation of the fluorescence intensity (Fig. 2 D, right panel). Specifically, while the fluorescence signal of Ig27-WT peaked at 338 nm, the p.Gly2939Ser-harboring Ig27 domain demonstrated a fluorescence maximum at 342 nm. Furthermore, Ig27-WT showed a significantly lower fluorescence amplitude than the p.Gly2939Ser-harboring Ig27 domain (190.7 ± 2.8 A.U. and 325.6 ± 11.9 A.U., respectively, *N* = 3, P < 0.001). Together, these results are indicative of a significant conformational change, in agreement with the structural modeling predictions (Fig. 2, A and B).

**Figure S1.**
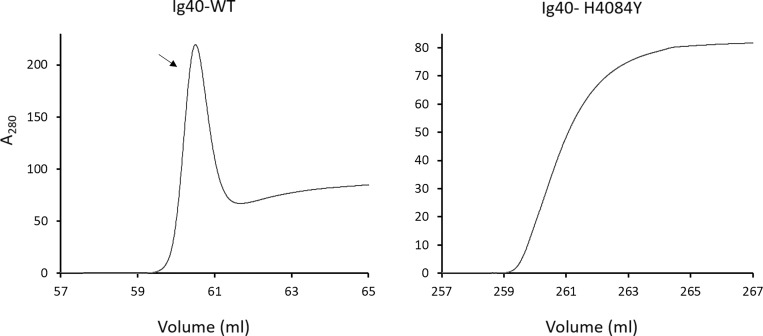
**Ig40 nickel exclusion chromatography. **Elution profile from nickel exclusion chromatography experiment for WT and mutant Ig40 domain. A peak corresponding to purified protein appears only in WT protein (black arrow). The result represents three independent experiments.

Constructs harboring Ig domains 38 and 40 could not be expressed in mammalian expression systems, prompting a shift to bacterial expression systems. While both the Ig38-WT and Ig40-WT domains were successfully expressed and purified (Fig. 2 E), the constructs bearing the p.Ala3969Thr or p.His4084Tyr variants showed minimal or no yield, respectively, under identical expression and purification conditions (Fig. 2 E and Fig. S1). Consistent with the structural modeling and MD simulations analyses, these results suggest that disruption of protein folding and stability is the mechanistic culprit underlying the pathogenicity of p.Ala3969Thr and p.His4084Tyr variants.

### Hemicentin-1 is mostly expressed in the human epidermis in the basal cell layer

**Figure S2.**
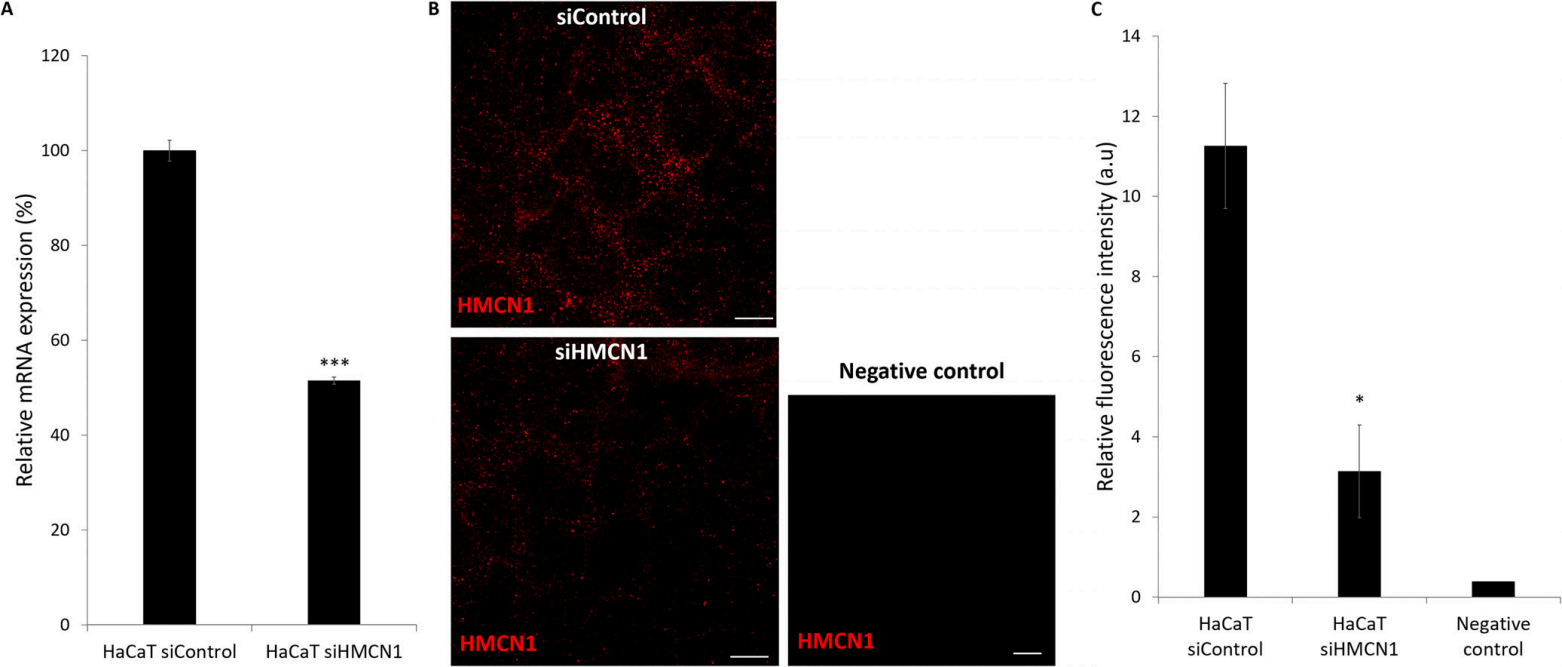
***HMCN1* antibody specificity. (A)** HaCaT cells were transfected with *HMCN1*-specific (HaCaT siHMCN1) or scramble (HaCat siControl) siRNAs. *HMCN1* mRNA levels were quantified using RT-qPCR. Results were normalized to *GAPDH* mRNA, represent the mean ± SE of two experiments, and are expressed as a percentage of *HMCN1* mRNA expression in cells transfected with siControl (two-sided *t* test; ***P < 0.005). **(B) **HaCaT cells were transfected with *HMCN1*-specific (siHMCN1) or scramble (siControl) siRNAs as described in A and immunostained using rabbit anti-hemicentin-1 antibody as described in Materials and methods. The negative control consisted of cells stained with the secondary antibody only. Note the decrease in hemicentin-1 staining in cells transfected with siHMCN1 (scale bar = 10 μm, hemicentin-1 [HMCN1] – red). **(C)** Fluorescence staining intensity in the experiment depicted in B was quantified by ImageJ. Result represents mean ± SE of two independent experiments (two-sided *t* test; *P < 0.05).

**Figure 3.**
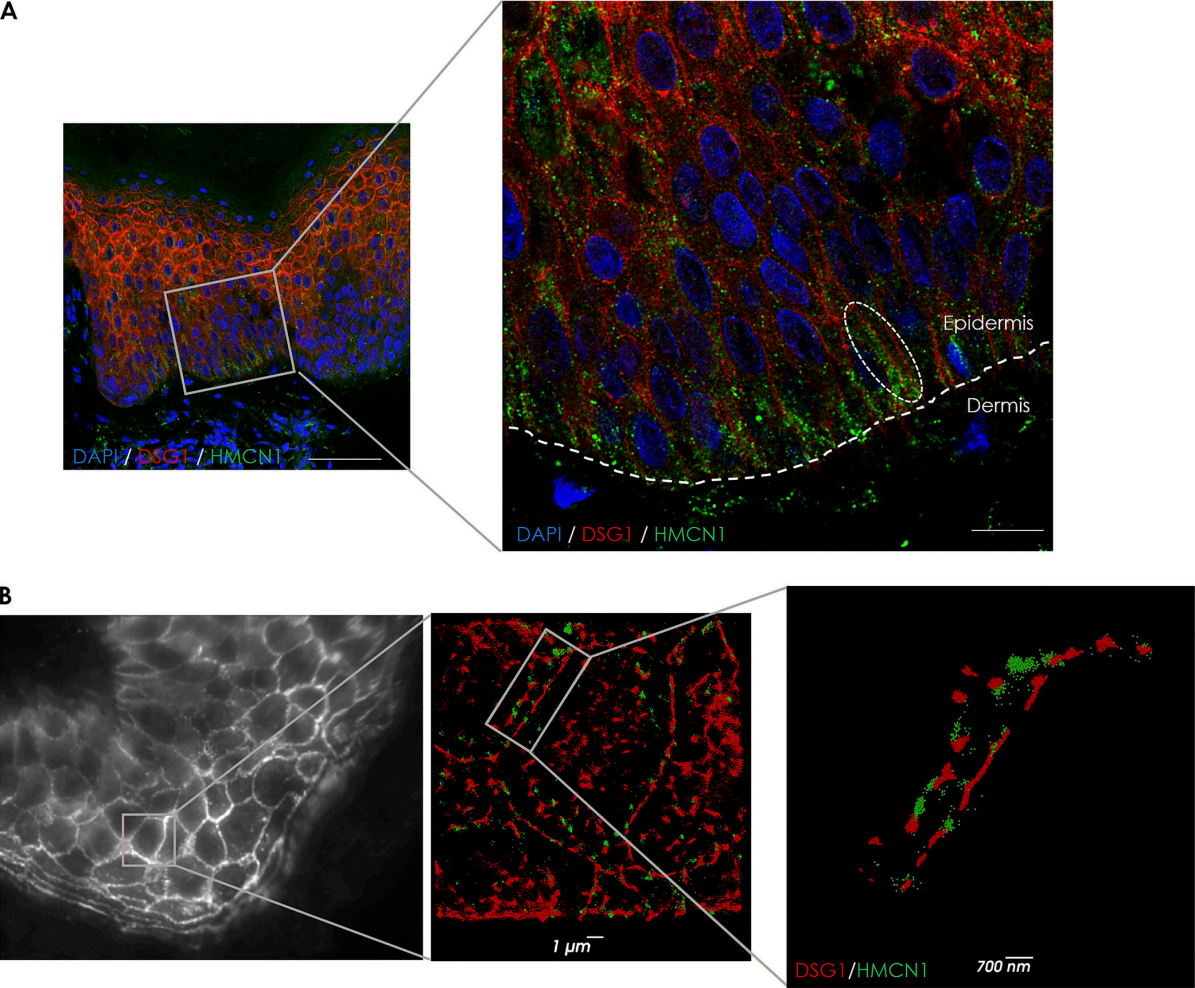
**Hemicentin-1 expression in the skin. (A)** Skin biopsies obtained from a healthy individual were co-immunostained using anti-hemicentin-1 and anti-desmoglein-1 antibodies. Scale bars = 100 μm (left panel) and 20 μm (right panel); hemicentin-1 (HMCN1), green; desmoglein-1 (DSG1), red; nuclei are stained in blue by DAPI; the experiment was repeated three times. **(B) **Super-resolution fluorescence microscopy (dSTORM) pictures of skin biopsies obtained from a healthy individual were co-immunostained using anti-hemicentin-1 and anti-desmoglein 1 antibodies (scale bars = 1 μm [middle panel] and 0.6 μm [right panel]; hemicentin-1 [HMCN1], green; desmoglein-1 [DSG1], red). See corresponding videos (Videos 1 and 2) (the experiment was repeated two times).

**Figure S3.**
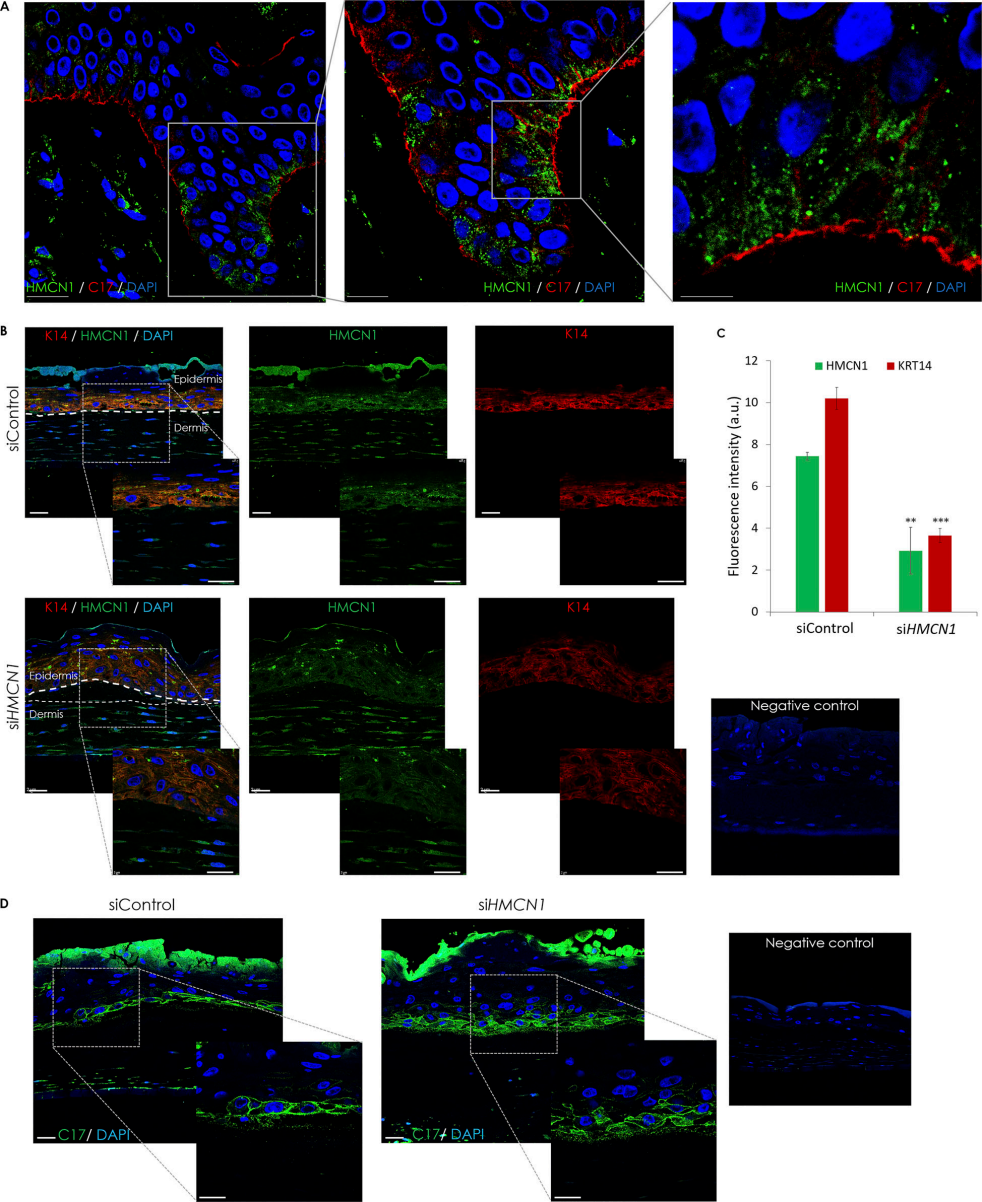
**Immunofluorescence studies in human skin and in three-dimensional skin equivalents. (A)** Skin sections obtained from a healthy individual were co-immunostained using anti- hemicentin-1 and anti-C17 antibodies (scale bars = 25 μm [left panel], 10 μm [middle panel], and 5 μm [right panel]; hemicentin-1 [HMCN1], green; C17, red; nuclei are stained in blue by DAPI). The experiment was repeated twice. **(B–D)** Human primary keratinocytes and fibroblasts transfected with *HMCN1*-specific siRNA (si*HMCN1*) or control siRNA (siControl) were used to generate three-dimensional organotypic cell cultures. **(B)** Punch biopsies were obtained from the skin equivalents at day 12, and co-stained for K14 and hemicentin-1 (HMCN1) (scale bar = 25 μm). **(C)** K14 and hemicentin-1 expression levels were quantified by ImageJ. Result represents mean ± SE of two independent experiments (one-way ANOVA; **P < 0.01, ***P < 0.005). **(D)** Human primary keratinocytes and fibroblasts transfected with *HMCN1*-specific siRNA (si*HMCN1*) or control siRNA (siControl) were used to generate three-dimensional organotypic cell cultures. Punch biopsies were obtained from the skin equivalents at day 12 and stained for C17 (scale bar = 25 μm). The experiment was repeated twice.

Given the fact that the three *HMCN1* variants associated with a more severe EBS phenotype were found to affect the hemicentin-1 structure, we hypothesized that (1) hemicentin-1 may contribute to the stability of the BMZ in human skin as previously shown in other species and tissues (Gianakas et al., 2023; Vogel and Hedgecock, 2001; Welcker et al., 2021; Zhang et al., 2022); and (2) that hemicentin-1 deficiency may compromise BMZ integrity and thus may aggravate the consequences of EBS-causing *KRT14* variants. Given that little is known about the localization of hemicentin-1 in human skin, we initially examined human skin sections double-stained for hemicentin-1 (using a specific anti-hemicentin 1 antibody, Fig. S2) and collagen 17 (C17) or desmoglein-1 by confocal microscopy. Hemicentin-1 was found to be expressed both in the epidermis and in the dermis. Although it was identified across the entire epidermis, it was much more significantly expressed at the basal cell layer, seemingly both within the cell cytoplasm and at the cell membrane (Fig. 3 A and Fig. S3 A).

To get a better understanding of the subcellular location of hemicentin-1, we used direct stochastic optical reconstruction microscopy (dSTORM). Super-resolution fluorescence microscopy clearly demonstrated that hemicentin-1 is mostly found at the cell membrane and less prominently in the intercellular space in the human epidermis (Fig. 3 B; and Videos 1 and 2).

**Video 1.** **A 360° view of the super-resolution fluorescence microscopy (dSTORM) analysis of normal skin sections co-immunostained with anti-hemicentin-1 and anti-desmoglein 1 antibodies (hemicentin-1 [HMCN1], green; desmoglein-1 [DSG1], red) as shown in** Fig. 3 B **(right panel).** Playback speed is 10 frames per second.

**Video 2.** **A similar view to the one shown in** Video 1 **but of a different position on the slide.** Playback speed is 10 frames per second.

### Hemicentin-1 directly interacts with K14

Given that (1) *HMCN1* variants associated with EBS severity affect protein domains likely to be responsible for regulating protein binding (Fig. 2), (2) human hemicentin-1 is expressed at the BMZ (Fig. 3), and (3) hemicentin-1 has been shown to bind various components of the BMZ and to play an important role in maintaining BMZ stability in other tissues and species (Gianakas et al., 2023; Welcker et al., 2021), we hypothesized that human hemicentin-1 binds components of the BMZ that are relevant to both BMZ integrity and EBS pathogenesis. Therefore, we attempted to identify hemicentin-1 binding partners in the skin.

**Figure 4.**
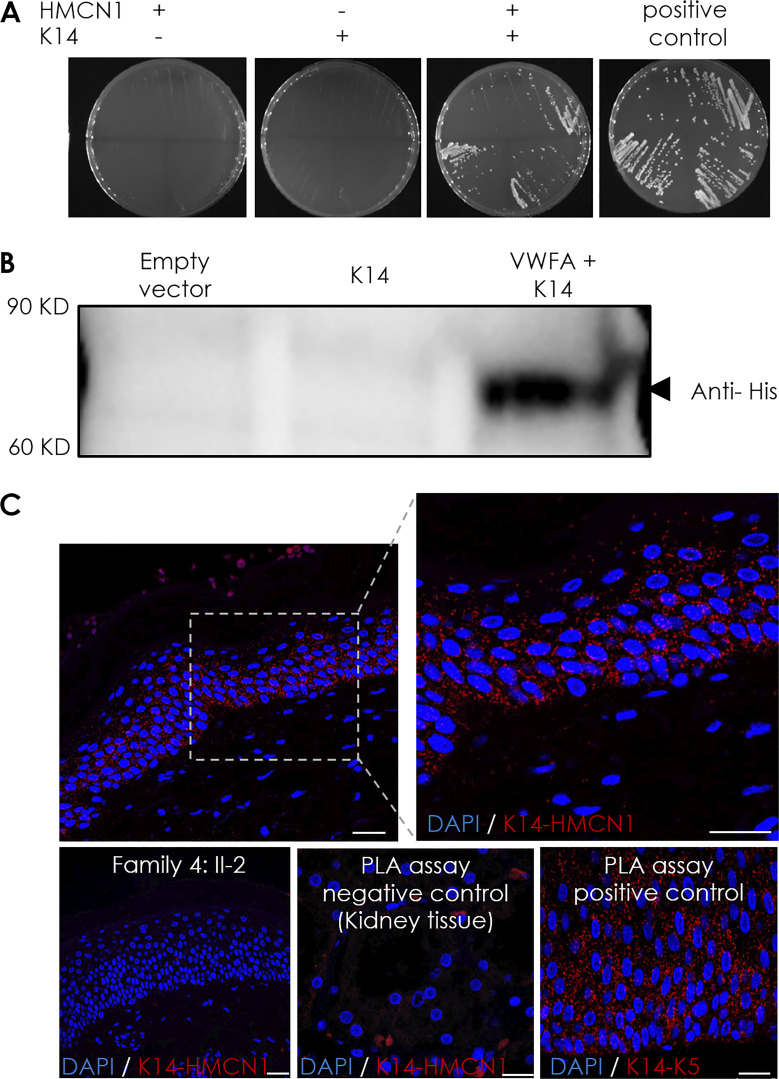
**Hemicentin-1 directly interacts with** **K** **14. (A)** A solid growth assay was used to validate hemicentin-1–K14 interaction identified in the Y2H assay. Yeasts were transfected with pB29-HMCN1 (aa 1–212, hemicentin-1) bait vector and pP7-KRT14 (aa 92–188, K14) prey vector. Yeast colonies were grown on DO-3 media (without tryptophan, leucine, and histidine), which selects for yeast harboring interacting bait and prey proteins. Note the presence of colonies in yeasts transfected with both constructs only. **(B)** Flag-tagged K14 and His-tagged hemicentin-1 VWFA domain-expressing plasmids were transfected into HeLa cells. Hemicentin-1 VWFA domain and K14 were co-immunoprecipitated using anti-Flag antibody-conjugated magnetic beads followed by immunoblotting of the precipitated proteins using anti-His antibody. The experiment was repeated three times with similar results. (**C)** PLA was performed using antibodies directed against hemicentin-1 and K14 in healthy human skin sections. Red dots indicate positive interaction (the experiment was repeated two times). The experiment was repeated using a skin biopsy taken from individual II-2, family 4 (scale bar, 50 μm), or kidney tissue a negative control. In addition, a PLA assay using antibodies directed against keratin 5 (K5) and K14 served as a positive control (scale bars, 25 μm). Source data are available for this figure: SourceData F4.

As the VWFA domain of hemicentin-1 is likely to mediate protein–protein interaction (Dong et al., 2006; Whittaker and Hynes, 2002), we cloned this domain to serve as a bait in a Y2H screen against a skin cDNA library. A number of proteins were identified as potential interacting partners of hemicentin-1, among which K14 stood out as *KRT14* variants are a major cause of EBS. We used a solid growth assay, as detailed in the Materials and methods section, to validate the results (Fig. 4 A).

**Figure S4.**
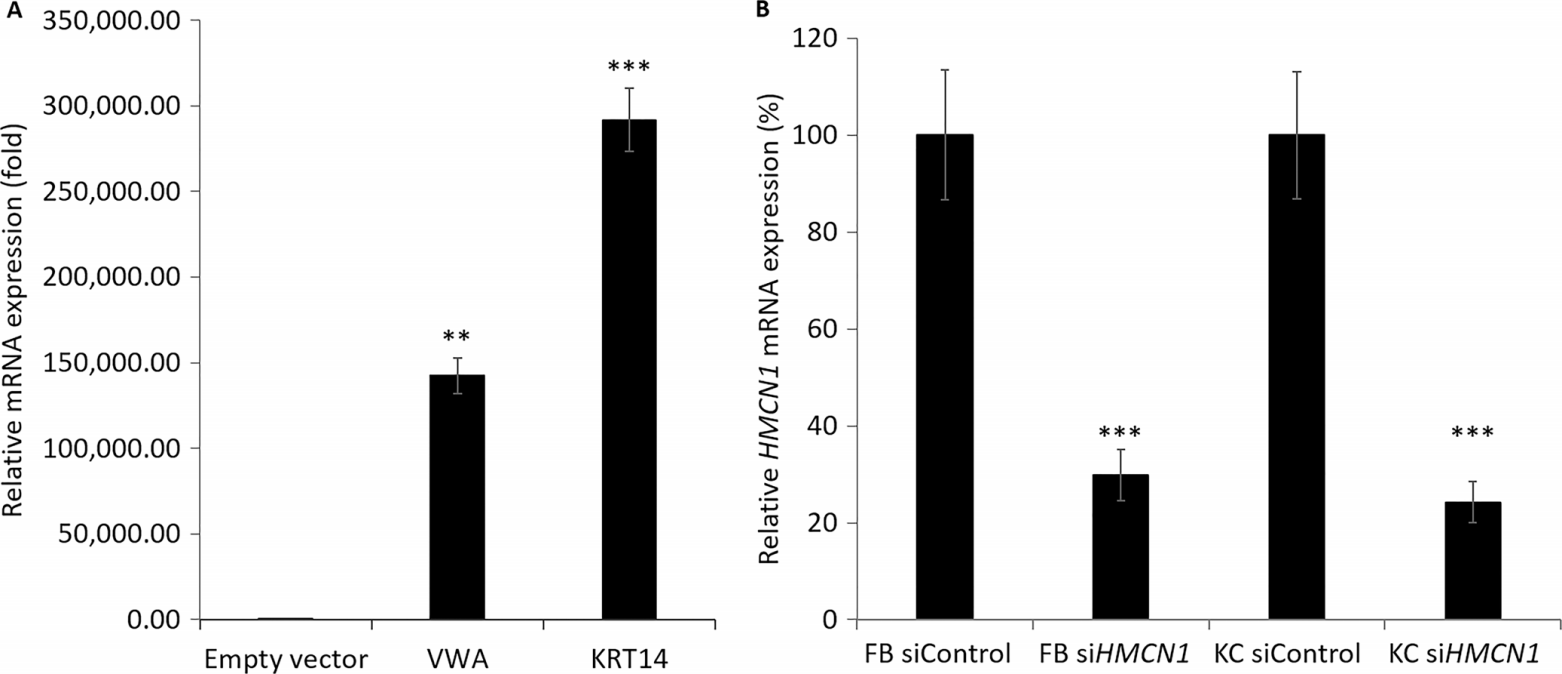
**Hemicentin-1 VWA domain, K14-encoding cDNA constructs expression in HeLa cells and *HMCN1* silencing efficiency. (A)** Hemicentin-1 VWA domain and K14 (KRT14)-expressing cDNA constructs were transfected in HeLa cells. Gene expression was quantified using RT-qPCR. Results were normalized to *GAPDH* mRNA, represent the mean ± SE of two experiments and are expressed as fold change of mRNA expression in cells transfected with an empty vector (one-way ANOVA; **P < 0.01, ***P < 0.005). **(B)***HMCN1* mRNA levels were quantified by RT-qPCR in primary human keratinocytes (KC) or fibroblasts (FB) transfected with *HMCN1*-specific (siHMCN1) or scramble (siControl) siRNAs. Results were normalized to *GAPDH* mRNA, represent the mean ± SE of three experiments, and are expressed as a percentage of *HMCN1* mRNA expression in cells transfected with siControl (two-way *t* test; ***P < 0.005).

To confirm in vitro the fact that hemicentin-1 interacts with K14, we co-transfected FLAG-tagged K14 and His-tagged hemicentin-1 VWFA domain plasmids (Fig. S4 A), co-immunoprecipitated proteins using anti-FLAG antibody, and immunoblotted the precipitated proteins using an anti-His antibody to identify the 75 kDa size VWFA domain protein. K14 and hemicentin-1 VWFA domains were found to co-precipitate (Fig. 4 B).

To substantiate these data in an *in vivo* context, we used a proximity ligation assay (PLA) assay using healthy human skin sections. The PLA assay is used to detect proteins expressed in close vicinity (<40 nm), one relative to the other (Hegazy et al., 2020). This assay confirmed the close proximity of hemicentin-1 and K14 in the epidermis (Fig. 4 C). No signal could be detected when the assay was performed using a biopsy from the kidney which does not express K14, or using a skin biopsy obtained from the patient II-2, family 4, indicative of a lack of interaction between K14 and hemicentin-1 (Fig. 4 C).

**Figure 5.**
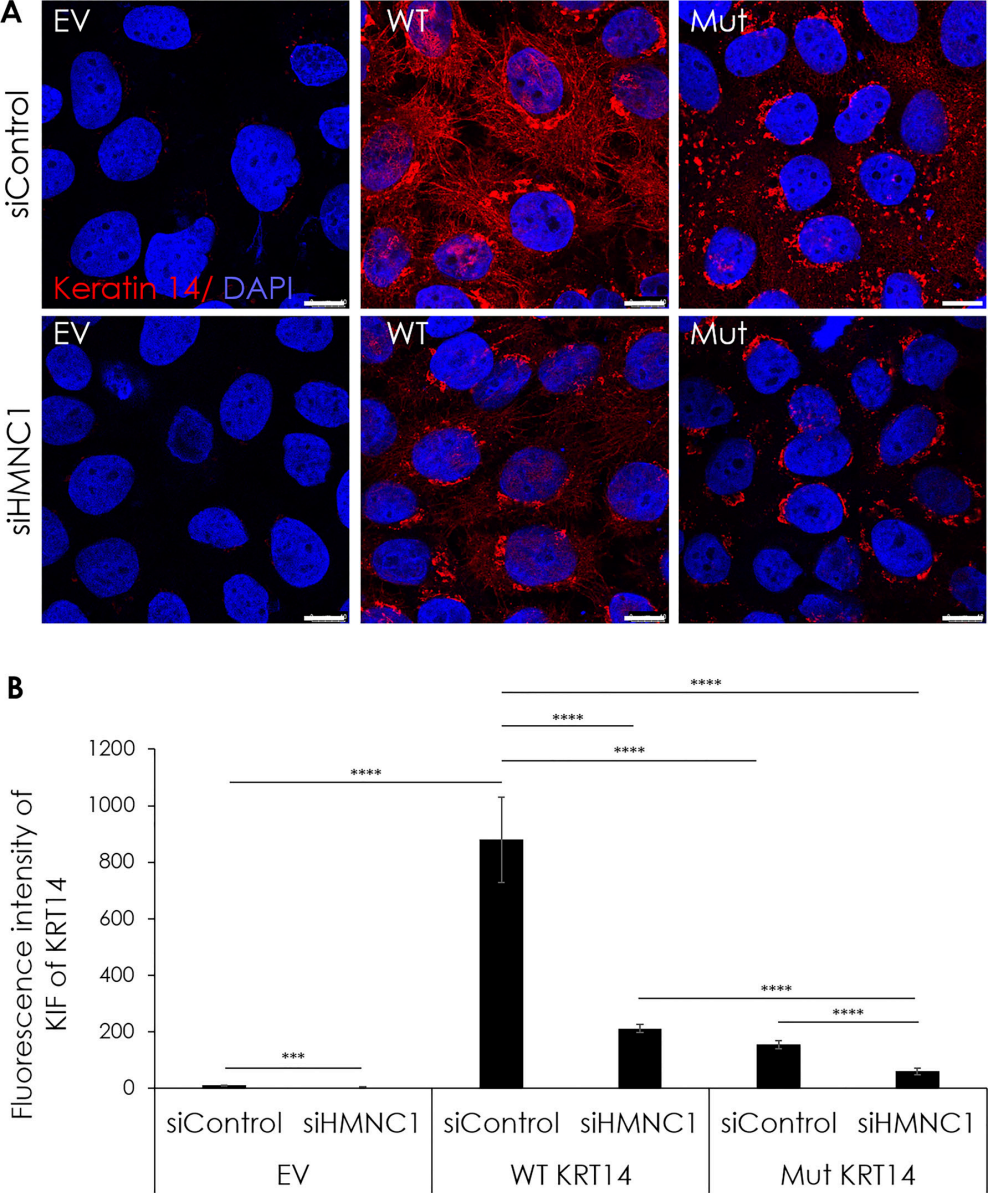
**Hemicentin-1 deficiency effect on KIF organization. (A)** HaCaT cells grown to 60% confluence on glass coverslips in 12 wells plates were downregulated for *HMCN1* with a specific siRNA (siHMCN1) or with a control siRNA (siControl) and co-transfected with an empty vector (EV) or expression vectors encoding either, WT cDNA, or mutant (Mut) *KRT14* cDNA carrying the c.373C>T, p.Arg125Cys variant for 48 h at 37°C. The cells were stained for *KRT14* expression (red staining) and DAPI (blue staining) (the experiment was repeated two times). Cells were visualized by confocal microscopy. Note protein aggregates in cells transfected with the mutant *KRT14* as well as lower expression levels of K14 in *siHMCN1*-transfected cells (scale bar = 10 μm). **(B)** K14 KIF fluorescence intensity per cell was measured using ImageJ, analyzing four different zones for each condition. Statistical analysis was performed by *t* test (***P < 0.005; ****P < 0.001).

**Figure S5.**
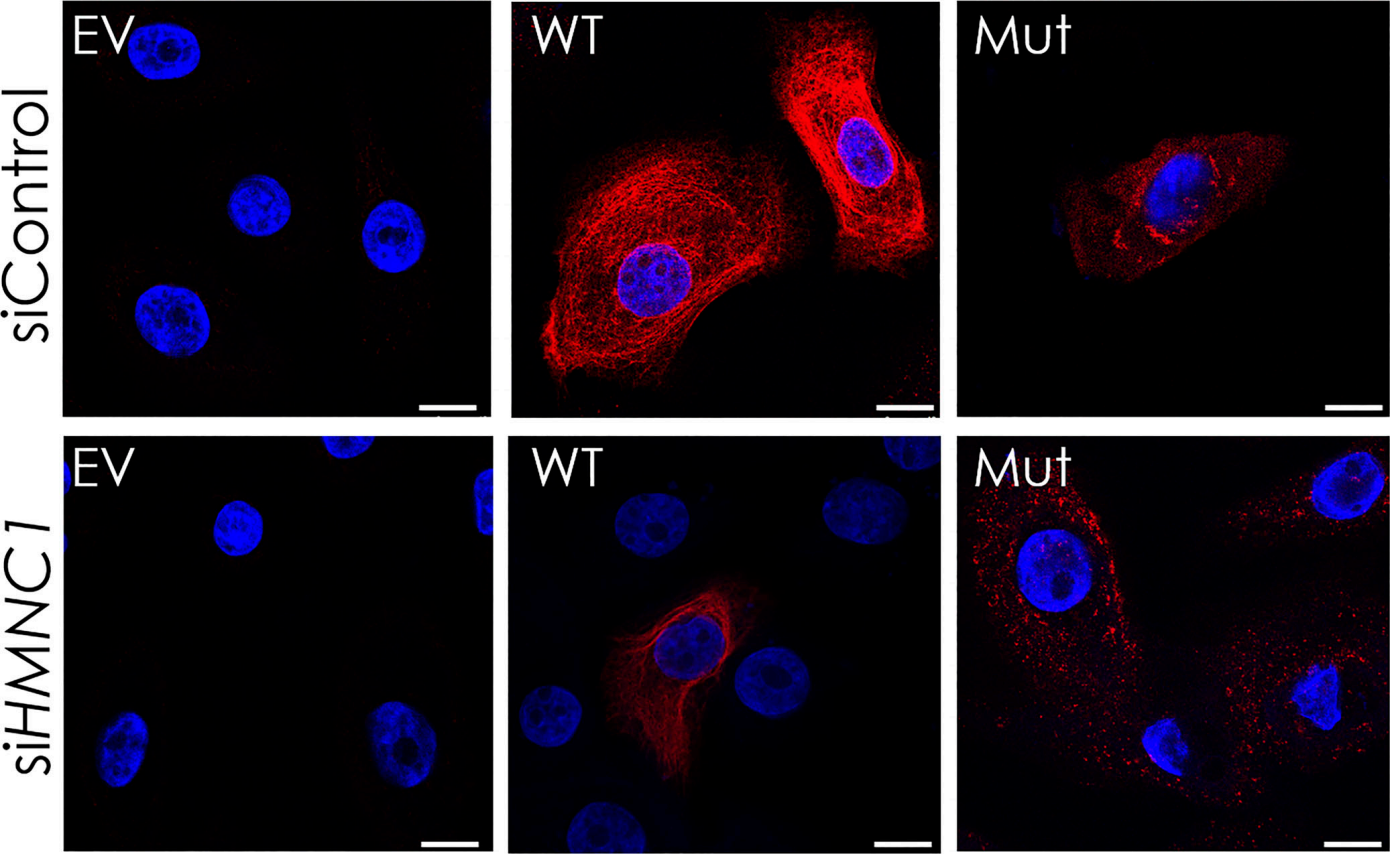
**Hemicentin-1 deficiency effect on KIF organization in primary keratinocytes.** Primary keratinocytes cells grown to 60% confluence on glass coverslips in 12 wells plates were downregulated for hemicentin 1 with a specific siRNA (siHMCN1) or with a control siRNA (siControl), and co-transfected with an empty vector (EV) or expression vectors encoding either WT cDNA or mutant (Mut) KRT14 cDNA carrying the c.373C>T, p.Arg125Cys variant for 48 h at 37°C. The cells were stained for KRT14 expression (red staining) and DAPI (blue staining). The experiment was repeated twice. Cells were visualized by confocal microscopy. Note decreased K14 expression upon *HMCN1* silencing and protein aggregates in cells transfected with the mutant KRT14, with marked aggravation in siHMCN1-transfected cells (scale bar = 10 μm).

Given the facts (1) that EBS results from abnormal organization of keratin intermediate filaments (KIF), (2) that hemicentin-1 interacts with K14 in the human epidermis, and (3) that hemicentins serve as scaffold proteins (Lin et al., 2020), we hypothesized that hemicentin-1 may be critical for proper KIF organization. We therefore ascertained the effect of hemicentin-1 deficiency on KIF organization. Keratinocytes transfected with mutant *KRT14* carrying the c.373C>T, p.Arg125Cys mutation identified in family 1, demonstrated decreased KIF organization associated with cytoplasmic clumping compared with cells transfected with the WT *KRT14*, as previously reported (Coulombe et al., 1991, 2009; Omary, 2009) (Fig. 5 and Fig. S5). *HMCN1* downregulation resulted in decreased KIF expression in keratinocytes transfected with WT *KRT14* and even more so in keratinocytes transfected with the mutant *KRT14* cDNA (Fig. 5 and Fig. S5), indicating that hemicentin-1 is critical for KIF integrity.

In addition, we conducted a proteomic analysis comparing control keratinocytes transfected with WT *KRT14* and control siRNA to keratinocyte cells downregulated for *HMCN1* and co-transfected with mutant *KRT14* carrying the c.373C>T, p.Arg125Cys mutation. This analysis revealed 84 proteins differentially expressed in *HMCN1* downregulated cells transfected with mutant *KRT14* (Table S3). Pathway analysis of the data revealed alteration in epidermis structural constituents, differentiation, and development as well as abnormal intermediate filament organization (Table S4). Of note, pathway analysis also revealed alteration in gap junction assembly, previously implicated in EBS (Liovic et al., 2009) (Table S4).

### Hemicentin-1 contributes to the stability of the epidermal**–**dermal junction

**Figure 6.**
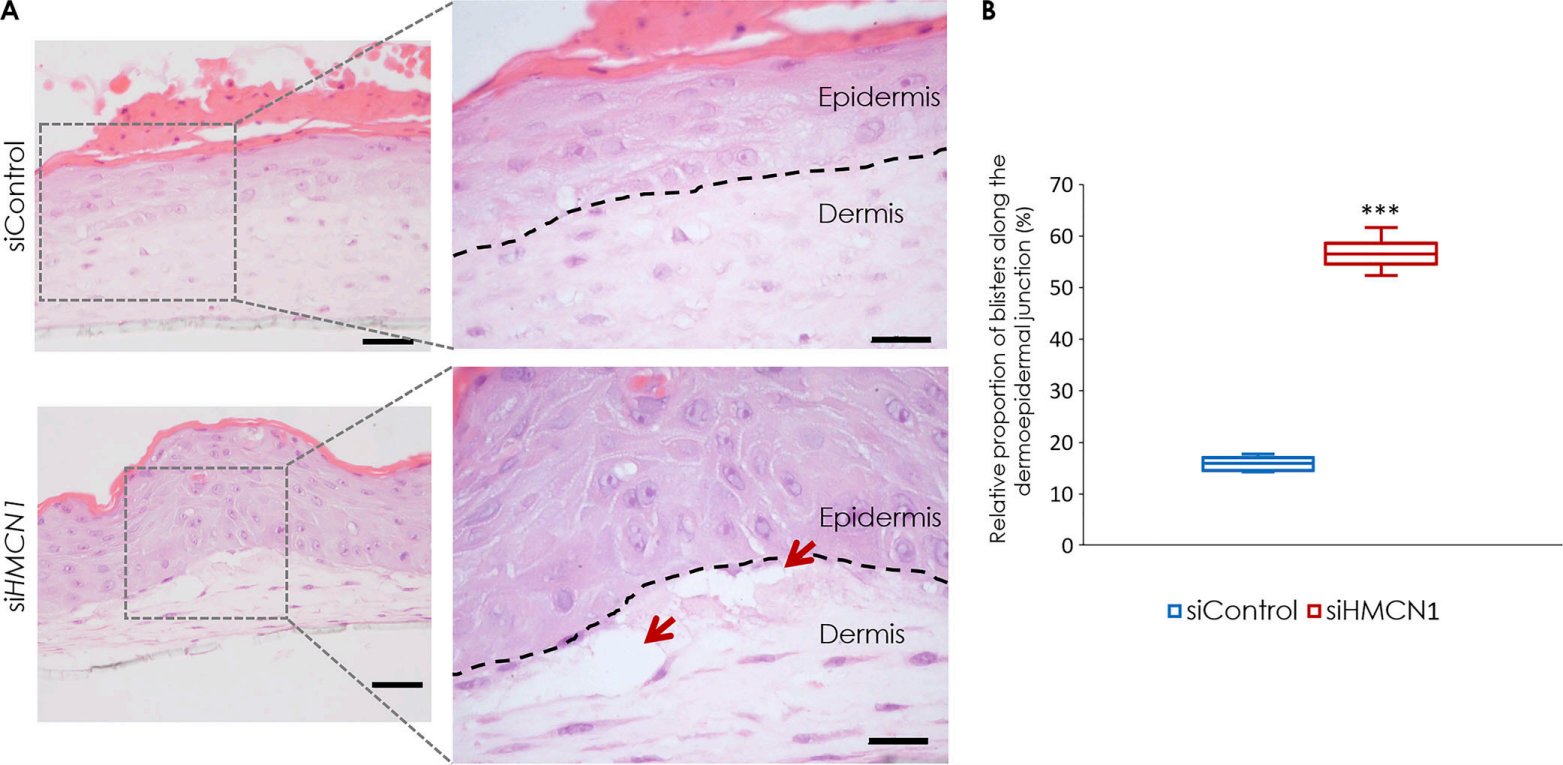
**Three-dimensional modeling of hemicentin-1 deficiency. (A)** Human primary keratinocytes and fibroblasts transfected with *HMCN1*-specific siRNA (siHMCN1) or control siRNA (siControl) were used to generate three-dimensional organotypic cell cultures. Punch biopsies were obtained from the skin equivalents at day 12 and stained for hematoxylin and eosin (scale bar, 50 μm). Red arrows indicate subepidermal blisters. **(B)** Subepidermal blistering was quantitatively ascertained as the ratio of the length of the blistering BMZ divided by the entire length of the BMZ using the NIS-Elements BR 3.2 software (Nikon). The plot represents the results of two independent experiments in which a total of 58 random fields were examined (two-sided *t* test; ***P < 0.005).

Given the fact that (1) deleterious variants in *HMCN1* are associated with severe EBS (Fig. 1 A) and (2) hemicentin-1 directly interacts with K14 (Fig. 4), we hypothesized that hemicentin-1 may contribute to the stability and integrity of the epidermal-dermal junction. To ascertain this possibility, we transfected normal keratinocytes and fibroblasts with control and *HMCN1*-specific siRNA (Fig. S4 B). We used those cells to generate three-dimensional organotypic cultures. This system faithfully replicates many central aspects of normal cutaneous biology and captures the true architecture of the skin (Poumay and Coquette, 2007). Control and hemicentin-1–deficient organotypic skin equivalents displayed normal differentiation. However, hemicentin-1–deficient organotypic skin equivalents consistently showed the spontaneous formation of subepidermal blisters after 12 days of culture (Fig. 6 A). Quantitative analysis of the blistering BMZ region (done by calculating the ratio of the length of the blistering BMZ divided by the entire length of the BMZ) revealed that areas of subepidermal separation were significantly more frequent in hemicentin-1–deficient skin equivalents (Fig. 6 B). Immunofluorescence microscopy revealed that hemicentin-1 is expressed both in the basal cell layer and in the dermis (Fig. S3, B and D) similar to what was seen in human skin sections (Fig. 3 A). Interestingly, *HMCN1* downregulation in the three-dimensional skin equivalents resulted in decreased K14 expression (Fig. S3, B and C). Taken collectively, these findings support a role for hemicentin-1 in the maintenance of a functional epidermal–dermal junction in human skin through interaction with K14 as evidenced by the decrease in K14 expression in hemicentin-1–deficient organotypic skin models.

## Discussion

Hemicentins are a family of extracellular matrix proteins first identified in *C. elegans*, with two orthologs (hemicentin-1 and 2) in vertebrates (Xu et al., 2013). To date, the function of hemicentin-1, also termed fibulin 6, in human skin has been poorly explored (Xu et al., 2013). Hemicentin-1 has been shown in various species to originate from epidermal as well as mesenchymal cells (Welcker et al., 2021). It has been shown in various cellular and animal models to play a number of roles including binding of basement membrane components and facilitation of BMZ assembly (Zhang et al., 2022; Gianakas et al., 2023; Morrissey et al., 2014), organization of epidermal hemidesmosomes (Vogel and Hedgecock, 2001), restriction of the stem cell compartment (Lindsay-Mosher et al., 2020), and regulation of cytokinesis (Xu and Vogel, 2011).

Hmcn1^−/−^ mice are viable but display ultrastructural BMZ alterations (Welcker et al., 2021), which is in agreement with the fact that human hemicentin-1–deficient skin equivalents show spontaneous subepidermal blister formation (Fig. 6), supporting a role for hemicentin-1 in BMZ maintenance.

To date, hemicentin-1 has been implicated in the pathogenesis of age-related macular degeneration, post-infarction myocardial fibrosis, cancer, and kidney glomerular disease (Chowdhury et al., 2014; Liu et al., 2019; Thompson et al., 2007; Toffoli et al., 2018). In addition, it has been proposed that hmcn1 and fbn2 interact functionally during in vivo zebrafish fin development to facilitate the correct formation of the fin dermo-epidermal junction (DEJ) and ensure proper tissue connection (Feitosa et al., 2012). Hmcn1 has been observed to co-localize with laminin-α2 at basement membranes surrounding hair follicles in the skin, particularly at the dermal–epidermal junctions (Welcker et al., 2021). The vertebrate extracellular matrix protein hemicentin-1 has been found to interact both physically and genetically with the basement membrane protein nidogen-2 (Zhang et al., 2022). In *C. elegans*, reduction of *vab-10a* (plakin) and loss of *him-4* (hemicentin) resulted in similar defects in BM–BM linkage, suggesting that they function together to link the gonadal and epidermal BMs under the anchoring cell (Morrissey et al., 2014).

Here, we show that deleterious variants in *HMCN1* co-segregate with a more severe phenotype in EBS and that hemicentin-1 contributes to the stability and integrity of the epidermal–dermal junction in human skin. We propose that hemicentin-1 may serve as a scaffold for various proteins, including K14, during the formation of the BMZ, as previously suggested in nematodes in other biological tissues (Morrissey et al., 2014; Xu et al., 2013). In the absence of hemicentin 1, KIF organization is defective, which may provide a mechanistic explanation for the more severe phenotype of EBS patients carrying both *HMCN1* and *KRT14* deleterious variants. Indeed, abnormal KIF formation is well-known to be causally associated with impaired epidermal–dermal adhesion (Seltmann et al., 2013, 2015).

The fact that EBS severity–associated variants are localized within the tandem Ig domains of hemicentin-1 underscores the central role these domains play in maintaining the structural and functional integrity of the protein and offers insights into the molecular mechanisms underlying phenotypic variability in EBS. Our novel finding of the interaction between hemicentin-1 N-terminal VWFA domain and K14 (Fig. 4), together with the established ability of hemicentin-1 C-terminal domains to interact with the basement membrane protein nidogen-2 (Zhang et al., 2022), is consistent with the role of hemicentin-1 in maintaining BMZ stability. From a structural perspective, hemicentin-1 is a very large protein mainly composed of tandem Ig domains, reminiscent of titin, which is composed of tandem Ig and fibronectin type-III domains (Linke, 2018). Intriguingly, the rigid and elongated arrangement of the tandem Ig domains of hemicentin-1 is similar to that reported for the tandem Ig domains of titin (Tskhovrebova et al., 2010). Within the striated muscle, titin interconnects the thick and thin filaments, spanning one-half of each sarcomere. Interestingly, reduced titin stiffness is associated with longer sarcomere length, and truncating mutations in titin result in dilated cardiomyopathy (Linke, 2018). Therefore, we propose that hemicentin-1 dysfunction contributes to abnormal skin function in a similar manner. Specifically, deleterious variants resulting in increased tandem Ig interdomain flexibility or reduced Ig domain stability hamper the ability of hemicentin-1 to simultaneously bind interacting proteins, which may then affect KIF organization (Fig. 5) and, ultimately, aggravate EBS phenotype. This model raises the possibility of therapeutic intervention in EBS by targeting keratin-interacting molecules rather than attempting to remove intracellular mutant keratin molecules, as previously proposed (Atkinson et al., 2011).

Apart from shedding light on the role of hemicentin-1 in human skin biology, our observations are of direct clinical relevance to the counseling of families at risk for EBS. Whether *HMCN1* variant analysis should become part of the molecular assessment of *KRT14*-associated EBS requires confirmatory studies. Of note, the wide range of basement membrane proteins that interact with hemicentin-1 (Xu et al., 2013) suggests that *HMCN1* variants may possibly modify the phenotypic expression of pathogenic variants associated with additional hereditary disorders of basement membranes (Jayadev and Sherwood, 2017) also associated with clinical variability (Bodemer et al., 2003) and in keratin variant-based diseases other than EBS (Pavlovsky et al., 2022).

## Materials and methods

### Patients

Patients were recruited at the Division of Dermatology, Tel Aviv Medical Center (Tel Aviv, Israel). Clinical and laboratory data were collected for all included patients. If possible, parents and siblings of the index case were also included. All participants or their legal guardians provided written and informed consent according to a protocol approved by the Tel Aviv Medical Center Institutional Review Board and by the Israeli National Committee for Genetic Studies in adherence to the Helsinki principles. The pictures used in this paper were taken with the consent of the patients and were approved by them for publication.

### Whole exome sequencing

Genomic DNA was extracted from peripheral blood leukocytes using the Gentra Puregene Blood Kit (Qiagen) according to the manufacturer’s instructions. Exome sequencing of patients III-1 and II-2, family 1; II-6, II-7, III-1, III-3 and III-5, family 2; I-1, I-2, II-1, II-2, II-3, II-4 and II-5, family 3; and II-2, family 4 (Fig. 1 A) was performed at the Tel Aviv Sourasky Genome Center. In short, targeted capture of protein-coding regions was performed using the IDT xGen capture reagent (Integrated DNA Technologies). Paired-end libraries were prepared from captured fragments and sequenced on the Illumina NovaSeq 6000 platform (Illumina). All exome sequencing data were analyzed using the Franklin by Genoox platform (https://www.Franklin.genoox.com). The next generation sequencing (NGS) pipeline used is based on the BWA aligner (Li and Durbin, 2009) and the two variant callers: GATK HaplotypeCaller (McKenna et al., 2010) and FreeBayes (Miller and Bishop, 2021). Rare variants were filtered using data from dbSNP155, the 1,000 Genomes Project, HGMD, gnomAD, Ensembl, Exome Variant Server, and an in-house database of individual exomes. Variants were classified by predicted protein and splicing effects using PolyPhen-2 (Adzhubei et al., 2010), SIFT (Kumar et al., 2009), Provean (Choi et al., 2012), ConSeq (Ashkenazy et al., 2010), Varsome (Kopanos et al., 2019), CADD (Rentzsch et al., 2021), SpliceAI (Jaganathan et al., 2019), BDGP (Reese et al., 1997), dbNSFP (Liu et al., 2011, 2016), and MutationTaster (Schwarz et al., 2014). Validation of the variants and co-segregation analysis were performed by direct sequencing.

### Direct sequencing

Genomic DNA was PCR-amplified using oligonucleotide primer pairs spanning the variants of interest (Table S5) with Taq polymerase (Qiagen). Cycling conditions were as follows: 94°C, 2 min; 94°C, 40 s; 61°C, 40 s; 72°C 100 s, for 3 cycles; 94°C, 40 s; 59°C, 40 s; 72°C 100 s, for 3 cycles; and 94°C, 40 s; 57°C, 40 s; 72°C 100 s, for 34 cycles. Gel-purified (QIAquick gel extraction kit; Qiagen) amplicons were subjected to bidirectional DNA sequencing as previously described (Malovitski et al., 2022). Sequencing results were analyzed using the Sequencher 5.4 software (Gene Codes).

### Quantitative real-time PCR (RT-qPCR)

For RT-qPCR, cDNA was synthesized from 1,000 ng of total RNA using a qScript kit (Quanta Biosciences). cDNA PCR amplification was carried out with the PerfeCTa SYBR Green FastMix (Quanta) on a StepOnePlus system (Applied Biosystems) with gene-specific intron-crossing oligonucleotides (Table S6) as previously described (Malovitski et al., 2022). Results were normalized to *GAPDH* mRNA levels.

### Cell cultures and reagents

Primary keratinocytes and fibroblasts were isolated from adult skin obtained from plastic surgery specimens after having received written informed consent from the donors according to a protocol reviewed and approved by the Tel Aviv Medical Center Institutional Review Board as previously described (Samuelov et al., 2013). Primary keratinocytes were maintained in a keratinocyte growth medium (Lonza). Fibroblasts were cultured in Dulbecco’s modified Eagle medium (DMEM) supplemented with 10% fetal calf serum (FCS; Biological Industries Israel), 50 U/ml penicillin, and 50 U/ml streptomycin (AppliChem). HeLa cells and human embryonic kidney 293 (HEK293) cells were maintained in DMEM 4.5 g/liter glucose medium containing 10% FCS, 1% L-glutamine, and 1% penicillin and streptomycin and grown at 37°C and 5% CO_2_ (Biological Industries). HaCaT cells were maintained in modified Eagle’s medium supplemented with 10% FCS, 1% L-glutamine, 1% streptomycin, and 1% amphotericin (Biological Industries).

### Gene silencing

Human keratinocytes or fibroblasts were cultured at 37°C in 5% CO_2_ in a humidified incubator. To downregulate *HMCN1* expression, we used human *HMCN1*-specific siRNA (sc-88362; Santa Cruz Biotechnology Inc.). As a control, we used Stealth RNAi siRNA Negative Control Lo GC (Invitrogen). Keratinocytes were transfected using 25 pmol of siRNA using Lipofectamine RNAiMax (Invitrogen).

### Organotypic cell cultures

Organotypic cell cultures were generated as previously described (Fuchs-Telem et al., 2011; Malovitski et al., 2022, 2023) using 0.25 × 10^6^ fibroblasts per ml of type I bovine collagen matrix (Advanced BioMatrix; PureCol) and 4 × 10^6^ keratinocytes per cm^2^ growth area, which were seeded onto 3-μm filter tissue culture inserts (BD). Models were grown for 12 days and the medium was changed every second day. For each set of experiments, keratinocytes and fibroblasts were derived from the same donor and used from the third passage. Punch biopsies were obtained from organotypic cell cultures and fixed in 4% paraformaldehyde. 5-μm-thick paraffin-embedded sections were processed for further staining.

### Immunofluorescence staining

For immunofluorescence studies of skin biopsies, antigen retrieval was performed as previously done (Malovitski et al., 2022; Mohamad et al., 2022). In short, 5-μm paraffin-embedded sections were deparaffinized using xylene/ethanol. Antigen retrieval was performed using 0.01 M citrate buffer, pH 6.0 (Invitrogen) in a microwave for 25 min, followed by blocking with 5% BSA in phosphate-buffered saline (PBS) for 45 min at room temperature. For double staining of hemicentin-1 and C17, sections were incubated with a rabbit anti-hemicentin-1 antibody (1:50, 18837-1-AP; Proteintech) overnight at 4°C, washed in PBS three times for 5 min, and incubated with a goat anti-rabbit IgG (H+L) cross-adsorbed secondary antibody, DyLight 488 (1:200, #35553; Invitrogen). Sections were then incubated with an Alexa-fluor 647 anti-C17 rabbit monoclonal antibody (1:100, ab194722; Abcam; Zotal Ltd.) overnight at 4°C and washed in PBS three times for 5 min. Coverslips were mounted with DAPI Fluoromount-G (Southern Biotechnologies). For double staining for hemicentin-1 and desmoglein-1, sections were co-incubated with a mix of rabbit anti-hemicentin-1 antibody (1:25, 18837-1-AP; Proteintech) and anti-desmoglein-1 mouse antibody (ready to use, Dsg1-P124; Progen) overnight at 4°C, washed in PBS three times for 5 min, and incubated with a mix of goat anti-rabbit IgG (H+L) cross-adsorbed secondary antibody, DyLight 488 (1:000, #35553; Invitrogen) and goat anti-mouse IgG (H+L) cross-adsorbed secondary antibody, and Rhodamine Red-X (1:000, #v; Invitrogen) for 60 min.

For cell immunofluorescence studies, HeLa cells were grown on glass coverslips and fixed with 4% paraformaldehyde. After permeabilization with 0.1% Triton/PBS, followed by blocking with 5% BSA in PBS for 60 min at room temperature, the cells were incubated with a mouse monoclonal 6XHIS antibody (1:1,000, MA1-21315; Invitrogen) overnight at 4°C, washed in PBS three times for 5 min, and incubated with Alexa Fluor 488 AffiniPure Donkey Anti-Mouse IgG (H+L) antibody (1:800, 715-545-150; Jackson) for 60 min. After three washes with PBS, the coverslips were incubated for 15 min in wells containing PBS with NucBlue Fixed Cell ReadyProbes Reagent (DAPI; Invitrogen) and after three additional washes with PBS, they were mounted using ProLong Gold Antifade Mountant (Invitrogen).

For HaCaT cells immunofluorescence studies, HaCaT cells were grown to 60% confluence on glass coverslips in 12-well plates and were downregulated for *HMCN1* with a specific siRNA (or control siRNA) and co-transfected with expression vectors encoding either WT or mutant *KRT14* (carrying the c.373C>T, p.Arg125Cys mutation) for 48 h at 37°C using Lipofectamine 2000 transfection reagent (Invitrogen). The cells were then fixed with 4% paraformaldehyde, blocked in 5% BSA, and incubated overnight at 4°C with a mouse monoclonal anti-KRT14 antibody (dilution 1:5; LL002; Bio Genex). Secondary antibody staining was carried out for 1 h at room temperature using Rhodamine RED anti-mouse antibody (Life Technologies/Invitrogen). Coverslips were mounted with DAPI Fluoromount-G (Southern Biotechnologies). Coverslips were mounted in polyvinyl alcohol. Staining was visualized using an LSM 700 confocal microscope (Carl Zeiss). Fluorescence intensity was quantified using ImageJ software.

### dSTORM

Sections were co-incubated with a mix of rabbit anti-hemicentin-1 antibody (1:25, 18837-1-AP; Proteintech) and anti-desmoglein 1 mouse monoclonal (Ready to use, Dsg1-P124; Progen) overnight at 4°C and washed in PBS three times for 5 min. The samples were then incubated with secondary antibodies as appropriate (anti-mouse Alexa Fluor 647, 1:500, ab150115; Abcam, and goat anti-rabbit CF568, 1:500, #20801; Biotium) for 120 min. After three washes with PBS, the samples were incubated in a switching buffer (50% glucose [10% in distilled water (DW)], 37.5% double DW, 10% 10xPBS, 2% cysteamine [5 M in DW], and 0.5% glucose oxidase [44,000 U/ml] with catalase [2,200 U/ml]) and sealed with nail polish.

*dSTORM* was conducted using a single-molecule localization microscope (Vutara 350; Bruker). We used 561- and 647-nm lasers with a power of 1,000 mW. The range of capabilities of our system are 5–10 kW/cm^2^. In this study, 30% laser power was applied (∼1.5–3 kW/cm^2^). A pentaband dichroic mirror and emissions filters were used throughout the study. The Vutara 350 custom case is designed for super-resolution, environmental isolation, temperature regulation, and drift minimization. The z-step between planes in the software is set to 100 nm. The cameras used are a sCMOS camera (4 MP, 6.5 × 6.5 μm pixel size for super-resolution imaging) and a CCD camera (1,392 × 1,040 for widefield imaging). All imaging was done using water immersion 60× objective (1.20 NA) on a field of view of 10 × 10 μm.

### Construct cloning, expression, and purification

A human *KRT14* cDNA construct harboring WT sequence or a mutant *KRT14* cDNA (c.373C>T, p.Arg125Cys) was cloned into pCDNA3.1 and labeled with DDDDK and V5 tags (Epoch Life Science, Inc.).

The N-(VWFA) terminal part of the human *HMCN1* cDNA sequence was cloned into pCDNA3.1 and labeled with 6XHis and V5 tags (Epoch Life Science).

Human *HMCN1* cDNA constructs harboring WT triple Ig domain (Ig27, Ig38 and Ig40, along with the two flanking Ig domains) cDNA sequence as well as mutant (c.8815G>A, c.11905G>A, c.12250C>T) sequences were cloned into pEZclone-NRS (Epoch Life Science) and then subcloned into paSHP-H mammalian expression vector modified with the BiP signal sequence and a C-terminal octa-histidine tag. For overexpression studies, the constructs were transiently transfected into HeLa cells grown to 80% confluence using Lipofectamine 3000 (Invitrogen). In addition, proteins were expressed in suspension-adapted HEK293 Freestyle cells (Invitrogen). Plasmid constructs were transfected into the cells using PEI MAX (Polysciences). The medium was harvested 7 days after transfection and secreted proteins were purified by nickel affinity chromatography.

For bacterial protein expression, *HMCN1* sequences coding for WT and mutant Ig38 domains of the human hemicentin-1 (residues 3897–3983) were cloned into pMAL expression vectors. Proteins were expressed in *E. coli* strain BL21 (DE3). Protein expression was induced at an OD_600_ of 0.5 with 1.0 mM isopropyl β-D-1-thiogalactopyranoside for 3 h at 37°C. The secreted proteins were purified from the periplasm. Cells were harvested by centrifuging and suspended in 0.5 M sucrose, 0.2 M Tris pH 8, 0.5 mM EDTA followed by adding 10% of the culture volume of ice-cold water, stirring, and centrifuging. Protein was purified by size-exclusion (Superdex 75 increase 10/300 GL column; Cytiva).

In addition, hemicentin-1 WT and mutant Ig40 domains-encoding sequences (residues 4079–4164) were cloned into pET28a expression vector, expressed in *E. coli* strain BL21 (DE3) and refolded from insoluble inclusion bodies as previously described (Zhang et al., 2002) with minor modifications. The refolding buffer consisted of 200 mM Tris pH 8.0, 10 mM EDTA, 0.5 M L-arginine, 1 mM L-Cysteine, and 1 mM L-Cystine. Ig40 domain protein refolded at 4°C was subjected to gel filtration chromatography on Superdex G-75.

### Yeast two-hybrid (Y2H) assay

Y2H assays were performed by Hybrigenics Services (Evry-Courouronnes) using the N-(VWFA) terminal part of the hemicentin-1 protein as bait for screening against an EpiSkin cDNA library as prey. In short, the coding sequence for human *HMCN1* (corresponding to amino acids 1–212) was PCR-amplified and cloned into pB29 as an N-terminal fusion to LexA (human HMCN1-LexA). The constructs were checked by sequencing the entire insert and used as bait to screen a random-primed human reconstituted skin cDNA library constructed into pP6. pB29 and pP6 are derived from the original pBTM116 (Béranger et al., 1997; Vojtek and Hollenberg, 1995) and pGADGH (Bartel et al., 1993) plasmids, respectively. 55.3 million clones (5.5-fold the complexity of the library) were screened using a mating approach with YHGX13 (Y187 ade2-101::loxP-kanMX-loxP, matα) and L40dGal4 (mata) yeast strains as previously described (Kabani et al., 2000). 81 His+ colonies were selected on a medium lacking tryptophan, leucine, and histidine. The prey fragments of the positive clones were amplified by PCR and sequenced at their 5′ and -3′ junctions. The resulting sequences were used to identify the corresponding interacting proteins in the GenBank database (NCBI) using a fully automated procedure. A confidence score (Predicted Biological Score) was attributed to each interaction as previously described (Formstecher et al., 2005).

### Solid growth tests

Solid growth tests were used to validate hemicentin-1–K14 protein interaction identified in the Y2H assay. Hemicentin-1 (aa 1–212, VWFA domain) was used as bait, and K14 (aa 92–188, EpiSkin_RP2_hgx5935v1_pB29_B-94) was used as prey. Bait and prey constructs were transformed in the yeast haploid cells L40deltaGal4 (mata) and YHGX13 (Y187 ade2-101::loxP-kanMX-loxP, matα), respectively. The diploid yeast cells were obtained using a mating protocol with both yeast strains (Fromont-Racine et al., 1997). These assays are based on the HIS3 reporter gene (growth assay without histidine). As negative controls, the bait plasmid was tested in the presence of an empty prey vector (pP7) and all prey plasmids were tested with the empty bait vector (pB27). The interaction between suppressor of mothers against decapentaplegic (SMAD) and SMAD-specific E3 ubiquitin protein ligase (SMURF) was used as the positive control (Colland et al., 2004). Controls and interactions were tested in the form of streaks of three independent yeast clones on DO-2 and DO-3 selective media. The DO-2 selective medium lacking tryptophan and leucine was used as a growth control and to verify the presence of the bait and prey plasmids. The DO-3 selective medium without tryptophan, leucine, and histidine selects for the interaction between bait and prey.

### Co-immunoprecipitation assay

Cells were transfected with expression vectors as described above for 48 h and then homogenized in IP Buffer (50 mM Tris, 150 mM NaCl, 5 mM EDTA, and 1% NP40) and a protease inhibitor mix (1:1,000. P8849; Sigma-Aldrich). Following centrifugation at 10,000 *g* for 10 min at 4°C, cell lysates were immunoprecipitated with the use of Pierce protein A/G magnetic beads (Invitrogen) according to the manufacturer’s instructions. In short, protein A/G magnetic beads were incubated, prior to immunoprecipitation, with a rabbit monoclonal anti-FLAG epitope tag antibody (10 μg/sample; Abcam) for 3 h, then washed three times using PBS + 0.1% Tween 20 (PBST), and immunoprecipitation was performed overnight at 4°C with the cell protein extract. The beads were then washed three times with PBST and the precipitated proteins were extracted from the beads into western blot sample buffer (X1) and incubated for 10 min at 70°C.

### Western blotting

Proteins were electrophoresed through a gradient Bio-Rad gel (4–20% Criterion TGX Stain-Free) and transferred onto a nitrocellulose membrane (Trans-Blot; Bio-Rad). After blocking for 1 h using 1 × Tris-buffered saline with Tween-20 (50 mM Tris, 150 mM NaCl, and 0.01% Tween 20) and 5% BSA, blots were incubated for 1 h at room temperature with a primary mouse monoclonal 6XHIS antibody (1:7,000, MA1-21315; Invitrogen) or mouse monoclonal antibody β-actin (1:5,000, ab8224; Abcam). The blots were washed five times for 5 min each with 1 × Tris-buffered saline and 0.1% Tween 20 with 1.5% BSA. After incubation with secondary horseradish peroxidase–conjugated goat anti-mouse antibody (diluted 1:10,000; Jackson Immuno Research Laboratories) and subsequent washings (five times, 5 min each with 1 × Tris-buffered saline Tween-20), proteins were detected using the Clarity Western ECL Substrate (Trans-Blot; Bio-Rad).

### PLA

PLA experiments were conducted on 5-μm paraffin-embedded skin sections as previously described (Hegazy et al., 2020). Sections were deparaffinized using xylene/ethanol and with Duolink In Situ Red Starter Kit Mouse/Rabbit (Sigma-Aldrich) according to the manufacturer’s instructions. We used the following antibodies: rabbit anti-hemicentin-1 antibody (1:25, 18837-1-AP; Proteintech), rabbit anti-Cytokeratin 5 monoclonal antibody (EP1601Y) (1:200, NB110-56916; Novus Biologicals), and mouse anti-cytokeratin 14 monoclonal antibody (LL002) (1:200, AM146; BioGenex).

### Structural modeling

The structures of Ig26–28 (residues 2766–2864), Ig 37–39 (residues 3804–4076), and Ig 39–41 (residues 3988–4255) of human hemicentin-1 (UniProt Q96RW7) were predicted using AlphaFold2 (Jumper et al., 2021) with the ColabFold pipeline (Mirdita et al., 2022). For each construct, five models were predicted and relaxed using amber, and the best-ranking model was used for further analyses. Models for the p.Gly2939Ser, p.Ala3969Thr, and p.His4084Tyr mutants were generated using Schrödinger Maestro release 2022–3 (Schrödinger). All the structures were prepared using the Protein Preparation Wizard. This protocol adds missing hydrogen atoms considering a pH value of 7.2 ± 1.0 and optimizes the hydrogen bond network. Next, energy minimization was performed using MacroModel (Schrödinger Release 2022–3: MacroModel) with the OPLS3e forcefield and Polack-Ribiere Conjugate Gradient algorithm. Minimization was stopped either after 2,500 steps of minimization or after reaching a convergence threshold of 0.05 kcal/mol. Structural representations were prepared using The PyMOL Molecular Graphics System Version 2.0 (Schrödinger, LLC).

### MD simulations

All the simulations were performed using the Schrödinger Maestro release 2022–3 (Schrödinger, LLC). The structures were prepared using the protein preparation wizard at a pH value of 7.2 ± 1.0 as described above. The system setup tool was used to solvate the systems using the TIP3P solvent model. Na^+^ or Cl^−^ ions were added to neutralize the charge and to obtain a final salt concentration of 150 mM. All MD simulations were performed using Desmond with the OPLS4 force field (Lu et al., 2021). The simulations were conducted under a Langevin temperature and pressure control using periodic boundary conditions with particle-mesh Ewald electrostatics with a 12 Å cutoff for long-range interactions. The systems were equilibrated using the default relaxation protocol and finally, the production simulations were carried out for 250 ns with a constant pressure of 1 atm and a constant temperature of 300 K. The results were manually inspected and analyzed using the Maestro suite.

### Protein stability prediction

All stability calculations were performed using FoldX suites (Schymkowitz et al., 2005) following the following protocol: Alphafold structural models were prepared using at least five rounds of the “RepairPDB” utility. Mutations were generated using the “BuildModel” utility and each calculation was repeated five times to test for convergence.

### Tryptophan fluorescence analysis

Fluorescence intensity spectra were recorded in a 100 μl quartz cuvette using a FP-8500 spectrophotometer (Jasco) at ambient room temperature. The protein concentration was 10 µM in PBS buffer. Fluorescence measurements were performed using sample excitation at 280 nm, with a 2.5-nm slit width, and the emission spectrum was recorded from 300 to 500 nm, using a 5-nm slit width. The spectrophotometer was configured with a 50-ms response time.

### Analytical size-exclusion chromatography

Protein samples were thawed, centrifuged at 21,000 *g* for 10 min, and loaded (75–150 mg) onto an analytical gel filtration column (Superdex 200 increase 10/300 GL column; Cytiva) pre-equilibrated with PBS buffer using an ultraperformance liquid chromatograph system (Shimadzu Corporation).

### Comparative proteomics analysis

HaCaT cells were grown to 60% in 6-well plates and downregulated for *HMCN1* with a specific siRNA (or control siRNA) and co-transfected with expression vectors encoding either WT or mutant *KRT14* (carrying the c.373C>T, p.Arg125Cys mutation) for 48 h at 37°C using Lipofectamine 2000 transfection reagent (Invitrogen).

Proteome analysis was performed at the Smoler Protein Research Center at the Technion, Haifa, Israel. Cells were lyzed in 8.5 M urea, 400 mM ammonium bicarbonate, and 10 mM DTT, sonicated twice (90%, 10–10, 5 min), and centrifuged (10,000 *g*, 10 min). The samples were reduced (60°C for 30 min), modified with 35.2 mM iodoacetamide in 100 mM ammonium bicarbonate (room temperature for 30 min in the dark), and digested in 1.5 M urea, 66 mM ammonium bicarbonate with modified trypsin (Promega), overnight at 37°C in a 1:50 (M/M) enzyme-to-substrate ratio. An additional second digestion with Trypsin was done for 4 h at 37°C in a 1:100 (M/M) enzyme-to-substrate ratio. The peptides were desalted using Oasis HLB 96-well µElution Plate (Waters), dried, and resuspended in 0.1% formic acid in 2% acetonitrile. The resulting peptides were analyzed by liquid chromatography with tandem mass spectrometry (LC-MS/MS) using an Exploris 480 mass spectrometer (Thermo Fisher Scientific) fitted with a capillary HPLC (Vanquish; Thermo Fisher Scientific). The peptides were loaded in solvent A (0.1% formic acid in water) on a homemade capillary column (30 cm, 75-μm ID) packed with Reprosil C18-Aqua (Dr. Maisch GmbH). The peptides mixture was resolved with a 5–28% linear gradient of solvent B (99.99% acetonitrile with 0.1% formic acid) for 180 min followed by a gradient of 15 min of 28–95% and 15 min at 95% solvent B at flow rates of 0.15 μl/min. Mass spectrometry was performed in a positive mode using repetitively full MS scan (m/z 380–985, resolution 120,000) followed by data-independent acquisition (DIA) scans (10 Da isolation windows with 1 m/z overlap and resolution 30,000).

The mass spectrometry data was analyzed using DIA-NN 1.8.1 (Demichev et al., 2020) for identification and quantification, searching against the human proteome from the Uniprot database. Protein N-terminus acetylation was accepted as variable modifications and carbamidomethyl on cysteine was accepted as static modifications. The identified proteins and peptides were filtered to a 1% false discovery rate. Statistical analysis of the identification and quantization results was done using Perseus 2.1.1.0 software (Tyanova et al., 2016).

Pathway analysis and clustering of differentially expressed protein was performed using STRING v12 (Szklarczyk et al., 2023) (https://string-db.org/) with a K-means algorithm with an average node degree score of 0.867 and average local clustering coefficient of 0.337.

### Statistical analysis

Comparisons of values between two groups were performed by the unpaired or paired Student’s *t* test. When more than two groups were evaluated, one-way ANOVA was performed. A value of P < 0.05 was considered statistically significant.

### Online supplemental material

Fig. S1 shows Ig40 nickel exclusion chromatography relating to Figs. 1 and 2. Additionally, Fig. S1 provides supporting data emphasizing the claims resulting from data in Fig. 2, A–D. Fig. S2 shows data that relates to Fig. 2, C–J, and supports *in vivo* observations. Fig. S3 shows flow cytometry data supporting the experiments described in Fig. 3. Fig. S4 shows hemicentin-1 VWA domain, K14-encoding cDNA constructs expression in HeLa cells, and *HMCN1* silencing efficiency. Fig. S5 shows hemicentin-1 deficiency effect on KIF organization in primary keratinocytes. Video 1 shows a 360° view of the super-resolution fluorescence microscopy (dSTORM) analysis of normal skin sections co-immunostained with anti-hemicentin-1 and anti-desmoglein 1 antibodies (hemicentin-1 [HMCN1], green; desmoglein-1 [DSG1], red) as shown in Fig. 3 B (right panel). Video 2 shows a similar view to the one shown in Video 1 but of a different position on the slide. Table S1 provides haplotype analysis in patients from families 3 and 4; Table S2 shows bioinformatics predictions of pathogenicity and number of healthy carriers; Table S3 lists differentially expressed proteins identified in the comparative proteomics analysis in cells downregulated for *HMCN1*; Table S4 provides pathway analysis and cluster analysis summary of the differentially expressed proteins identified in Table S3; Table S5 list oligonucleotides used for *HMCN1* direct sequencing; Table S6 list oligonucleotides used for RT-qPCR.

## Supplementary Material

Table S1 shows haplotype analysis in patients from families 3 and 4 carrying the *KRT14* c.1163G>A; p.Arg388His variant.

Table S2 shows bioinformatic predictions of pathogenicity and number of healthy carriers.

Table S3 list of differentially expressed proteins identified in the comparative proteomics analysis in cells downregulated for *HMCN1*.

Table S4 shows pathway analysis and cluster analysis summary of differentially expressed proteins identified in the comparative proteomics analysis (Table S3).

Table S5 shows the sequence of oligonucleotides used for *HMCN1* direct sequencing.

Table S6 shows the sequence of oligonucleotides used for RT-qPCR.

SourceData F2 is the source file for Fig. 2.

SourceData F4 is the source file for Fig. 4.

## Data Availability

Full exome sequencing data are not available to protect patient privacy; anonymized variant data will be made available upon reasonable request. The authors declare that all other data are contained within the manuscript and supplemental materials.
