# Supplementary material for: HMCN1 variants aggravate epidermolysis bullosa simplex phenotype
Source: J Exp Med. 2025 Feb 20;222(5):e20240827. doi: 10.1084/jem.20240827 (PMC11841684; doi:10.1084/jem.20240827)
Supplement: Table S1 — shows haplotype analysis in patients from families 3 and 4 carrying the KRT14 c.1163G>A; p.Arg388His variant. [file jem_20240827_tables1.docx]

**Table S1. Haplotype analysis in patients from families 3 and 4 carrying the *KRT14* c.1163G>A; p.Arg388His variant**

| **Locus** | **Chromosomal position** | **Family 4**  **II-1** | | **Family 3**  **II-1** | | **Family 3**  **II-2** | | **Pathogenic variant** |
| --- | --- | --- | --- | --- | --- | --- | --- | --- |
| rs60125824 | 17:39,645,003 | G | G | C | G | C | G |  |
| rs61126695 | 17:39,659,091 | C* | C | C | C | C | C |  |
| rs73986307 | 17:39,660,005 | C | C | C | C | C | C |  |
| rs760134 | 17:39,661,366 | C | C | C | C | C | C |  |
| rs2305556 | 17:39,671,724 | C | C | C | C | C | C |  |
| rs3744784 | 17:39,674,540 | G | G | G | G | G | G |  |
| rs58645163 | 17:39,739,598 | T | T | T | T | T | T | c.1163G>A |
| rs3826550 | 17:39,742,807 | T | T | T | T | T | T |  |
| rs6503639 | 17:39,742,856 | A | A | A | A | A | A |  |
| rs117941474 | 17:39,775,870 | T | T | T | T | T | T |  |
| rs9916519 | 17:39,779,160 | C | C | C | C | C | C |  |
| rs35612698 | 17:39,884,065 | A | A | A | A | A | A |  |
| rs3809877 | 17:39,959,511 | T | T | C | T | C | T |  |

*The shared haplotype is colored in blue
