# Supplementary material for: HMCN1 variants aggravate epidermolysis bullosa simplex phenotype
Source: J Exp Med. 2025 Feb 20;222(5):e20240827. doi: 10.1084/jem.20240827 (PMC11841684; doi:10.1084/jem.20240827)
Supplement: Table S2 — shows bioinformatic predictions of pathogenicity and number of healthy carriers. [file jem_20240827_tables2.docx]

**Table S2. Bioinformatic predictions of pathogenicity and number of healthy carriers**

| DNA variant, protein variant | GERP++ (range -12.3-6.17) | PROVEAN (range 14-(-) 14) | SIFT4G (range 1-0) | PolyPhen2 (range 0-1) | CADD (range 0-99) | Number of healthy carriers identified** |
| --- | --- | --- | --- | --- | --- | --- |
| c.8815G>A, p.Gly2939Ser | 5.44* | -2.98 | 0.007 | 0.987 | 27.3 | 14 |
| c.11905G>A, p.Ala3969Thr | 5.59 | -3.11 | 0 | 1 | 28.2 | 78 |
| c.12250C>T, p.His4084Tyr | 5.95 | -2.06 | 0.003 | 0.986 | 23 | 93 |

* Deleterious/Benign

** Out of 8,011 healthy people
