# Supplementary material for: HMCN1 variants aggravate epidermolysis bullosa simplex phenotype
Source: J Exp Med. 2025 Feb 20;222(5):e20240827. doi: 10.1084/jem.20240827 (PMC11841684; doi:10.1084/jem.20240827)
Supplement: Table S3 — list of differentially expressed proteins identified in the comparative proteomics analysis in cells downregulated for HMCN1. [file jem_20240827_tables3.docx]

**Table S3. List of differentially expressed proteins identified in the comparative proteomics analysis in cells down regulated for *HMCN1* (or control) and carrying mutant *KRT14*.** The analysis was performed on three independent experiments.

| # | Gene name | p-value | Difference in protein expression (log2) |
| --- | --- | --- | --- |
| 1 | PI3 | 0.000063 | 6.395 |
| 2 | KCTD2 | 0.000071 | 4.234 |
| 3 | DCAF4 | 0.040791 | 3.308 |
| 4 | SLC36A4 | 0.034264 | 2.865 |
| 5 | TUBB4A | 0.005042 | 2.628 |
| 6 | KCNK10 | 0.006664 | 1.513 |
| 7 | FAM89B | 0.036932 | 1.496 |
| 8 | HACD4 | 0.013661 | 1.305 |
| 9 | NWD2 | 0.037742 | 1.164 |
| 10 | ITIH4 | 0.032667 | 1.162 |
| 11 | FST | 0.014277 | 1.115 |
| 12 | RELT | 0.001231 | 1.077 |
| 13 | CYB561D2 | 0.029445 | 1.057 |
| 14 | THBS1 | 0.005844 | 1.052 |
| 15 | IGFBP3 | 0.000500 | 0.923 |
| 16 | STING1 | 0.025958 | 0.916 |
| 17 | GABARAP | 0.001246 | 0.911 |
| 18 | GJA1 | 0.005522 | 0.890 |
| 19 | SPRR3 | 0.004598 | 0.889 |
| 20 | GJB3 | 0.000187 | 0.880 |
| 21 | TCTN3 | 0.018866 | 0.872 |
| 22 | SNX19 | 0.004446 | 0.869 |
| 23 | PACC1 | 0.009410 | 0.846 |
| 24 | ZNF808 | 0.010403 | 0.839 |
| 25 | CTSV | 0.038612 | 0.809 |
| 26 | KRT23 | 0.004859 | 0.804 |
| 27 | SPRR1A | 0.005020 | 0.801 |
| 28 | ZNF142 | 0.015833 | 0.790 |
| 29 | WWC2 | 0.016114 | 0.752 |
| 30 | HMGCS1 | 0.010701 | 0.750 |
| 31 | SH2D5 | 0.025974 | 0.745 |
| 32 | UBE2E3 | 0.041233 | 0.742 |
| 33 | CDC7 | 0.006696 | 0.741 |
| 34 | F3 | 0.008874 | 0.735 |
| 35 | KRT36 | 0.014186 | 0.718 |
| 36 | RRN3 | 0.018680 | 0.713 |
| 37 | ARHGAP11A | 0.003546 | 0.711 |
| 38 | NUDT13 | 0.037861 | 0.703 |
| 39 | SPRR1B | 0.006790 | 0.702 |
| 40 | SASS6 | 0.006081 | 0.699 |
| 41 | CRIM1 | 0.012807 | 0.690 |
| 42 | FOSL1 | 0.032329 | 0.680 |
| 43 | MMADHC | 0.029536 | 0.679 |
| 44 | SPHK1 | 0.007691 | 0.670 |
| 45 | ZNHIT3 | 0.044951 | 0.668 |
| 46 | MAP1LC3B | 0.022304 | 0.662 |
| 47 | WRAP73 | 0.008086 | 0.661 |
| 48 | KCNK1 | 0.002232 | 0.656 |
| 49 | MTHFD2 | 0.010510 | 0.656 |
| 50 | TMEM154 | 0.003421 | 0.641 |
| 51 | ETS1 | 0.023270 | 0.633 |
| 52 | APEX2 | 0.023806 | 0.632 |
| 53 | ERRFI1 | 0.014695 | 0.631 |
| 54 | PLAUR | 0.001461 | 0.625 |
| 55 | PRKAR1B | 0.028246 | 0.607 |
| 56 | MRGPRX2 | 0.029765 | 0.604 |
| 57 | IL7R | 0.026197 | 0.601 |
| 58 | LRP10 | 0.009879 | 0.600 |
| 59 | SLCO4A1 | 0.016133 | 0.597 |
| 60 | RBM18 | 0.043752 | 0.595 |
| 61 | PCLAF | 0.015070 | 0.594 |
| 62 | AXIN1 | 0.021453 | 0.589 |
| 63 | PABIR2 | 0.042331 | -0.585 |
| 64 | HPGD | 0.043528 | -0.618 |
| 65 | FOXO1 | 0.003350 | -0.631 |
| 66 | SETDB2 | 0.004088 | -0.636 |
| 67 | RFNG | 0.003793 | -0.680 |
| 68 | LYSMD1 | 0.026027 | -0.693 |
| 69 | TADA2A | 0.031698 | -0.725 |
| 70 | GMEB1 | 0.003118 | -0.725 |
| 71 | KRT74 | 0.046715 | -0.785 |
| 72 | NIPSNAP3A | 0.012922 | -0.791 |
| 73 | HSH2D | 0.000099 | -1.086 |
| 74 | PHC1 | 0.017200 | -1.157 |
| 75 | S100A7 | 0.047063 | -1.172 |
| 76 | KDM4D | 0.045509 | -2.404 |
| 77 | TNFSF9 | 0.039636 | -3.104 |
| 78 | NATD1 | 0.049358 | -3.352 |
| 79 | VHL | 0.039262 | -4.174 |
| 80 | HCAR2 | 0.049770 | -5.137 |
| 81 | TINF2 | 0.042860 | -5.379 |
| 82 | PADI3 | 0.000032 | -5.540 |
| 83 | PRODH | 0.000032 | -6.969 |
| 84 | HOXC12 | 0.000005 | -7.252 |
