# Supplementary material for: HMCN1 variants aggravate epidermolysis bullosa simplex phenotype
Source: J Exp Med. 2025 Feb 20;222(5):e20240827. doi: 10.1084/jem.20240827 (PMC11841684; doi:10.1084/jem.20240827)
Supplement: Table S4 — shows pathway analysis and cluster analysis summary of differentially expressed proteins identified in the comparative proteomics analysis (Table S3). [file jem_20240827_tables4.docx]

**Table S4. Pathway analysis and cluster analysis summary of differentially expressed proteins identified in the comparative proteomics analysis (Table S3).**

Pathway analysis and clustering of differentially expressed proteins were performed using STRING with a K-means algorithm.

| **Clusters and term description** | **p-value** |
| --- | --- |
| **Cluster 1 - Formation of the cornified envelope** |  |
| Epithelial cell differentiation | 3.77E-06 |
| Peptide cross-linking | 8.79E-06 |
| Epidermis development | 0.00025 |
| Keratinocyte differentiation | 0.00055 |
| Intermediate filament organization | 0.0045 |
| Keratinization | 0.0069 |
| Structural molecule activity | 5.80E-06 |
| Structural constituent of skin epidermis | 0.0011 |
| Cornified envelope | 1.35E-05 |
| Cytosol | 0.0347 |
| Intermediate filament | 0.0438 |
| Epidermal cell | 7.41E-08 |
| Keratinocyte | 3.84E-07 |
| Cornified envelope | 4.54E-06 |
| Keratin, type I | 0.0021 |
| Intermediate filament protein, conserved site | 0.0068 |
| Intermediate filament, rod domain | 0.0073 |
| Intermediate filament protein | 0.0023 |
| **Cluster 2 - Regulation of plasminogen activation** |  |
| Regulation of blood coagulation | 0.0152 |
| Regulation of endopeptidase activity | 0.0152 |
| Regulation of plasminogen activation | 0.0287 |
| Regulation of fibrinolysis | 0.0287 |
| Negative regulation of endopeptidase activity | 0.0404 |
| Positive regulation of blood coagulation | 0.0404 |
| Response to cytokine | 0.0404 |
| Response to progesterone | 0.0494 |
| **Cluster 3 - Gap junction assembly** |  |
| Connexin complex | 0.029 |
| Connexin, conserved site | 0.0214 |
| Gap junction assembly | 0.00018 |
| Membrane Trafficking | 0.0029 |
| Microtubule-dependent trafficking of connexons from Golgi to the plasma membrane | 0.0041 |
| Patchy palmoplantar hyperkeratosis | 0.0226 |
| Erythrokeratodermia variabilis | 0.0035 |
| Connexin complex | 0.0181 |
| Gap junction | 0.014 |
| Palmoplantar keratoderma | 0.014 |
| Connexin homologues | 0.0098 |
| Gap junction channel protein cysteine-rich domain | 0.0098 |
| **Cluster 4 - Mitotic sister chromatid segregation and Mitotic spindle checkpoint sign** |  |
| Mitotic sister chromatid segregation, and Mitotic spindle checkpoint signaling | 0.046 |
| **Cluster 5 - Tandem pore domain potassium channels** |  |
| Stabilization of membrane potential | 0.0138 |
| Potassium ion leak channel activity | 0.0039 |
| Voltage-gated potassium channel activity | 0.0327 |
| Tandem pore domain potassium channels | 0.0018 |
| Tandem pore domain potassium channels | 0.0011 |
| Phase 4 - resting membrane potential | 0.0012 |
| Potassium channel | 0.0104 |
| Ion channel | 0.0256 |
| Two pore domain potassium channel | 0.005 |
| Potassium channel domain | 0.005 |
