# Supplementary material for: HMCN1 variants aggravate epidermolysis bullosa simplex phenotype
Source: J Exp Med. 2025 Feb 20;222(5):e20240827. doi: 10.1084/jem.20240827 (PMC11841684; doi:10.1084/jem.20240827)
Supplement: Table S5 — shows the sequence of oligonucleotides used for HMCN1 direct sequencing. [file jem_20240827_tables5.docx]

**Table S5. Sequence of oligonucleotides used for *HMCN1* direct sequencing**

| Exon | Forward  oligonucleotide sequence | Reverse  oligonucleotide sequence | Expected product size (bp) |
| --- | --- | --- | --- |
| 57 | GATGATTCTTCCGGATGTGGC | GGTGTGCTGGTGGTTTTAAAGG | 1137 |
| 78 | GCTAAGGCCAGGGTTGTGAG | GCTCTCGACAGTACCTACCACC | 556 |
| 81 | TGATATGGTGAAGTCAGGATTTTG | CACTTTGGATTACTGAGTTTAAGGG | 509 |
