# Supplementary material for: HMCN1 variants aggravate epidermolysis bullosa simplex phenotype
Source: J Exp Med. 2025 Feb 20;222(5):e20240827. doi: 10.1084/jem.20240827 (PMC11841684; doi:10.1084/jem.20240827)
Supplement: Table S6 — shows the sequence of oligonucleotides used for RT-qPCR. [file jem_20240827_tables6.docx]

**Table S6. Sequence of oligonucleotides used for RT-qPCR**

| Gene | Forward  oligonucleotide sequence | Reverse  oligonucleotide sequence |
| --- | --- | --- |
| *KRT14* | GCGGCTGGAGCAGGAGAT | GGAGGAGGTCACATCTCTGGAT |
| *GAPDH* | GAGTCAACGGATTTGGTCGT | GACAAGCTTCCCGTTCTCAGCC |
| *HMCN1* | GCTGATGGTAGTCTGTATGTGG | TCCTCGTTGATCTCCAAACAC |
| *HMCN1 Ig26-Ig27* | CGTGCCGCCAATTATCAAGG | AGGGCTGTCCATCTTTCTGC |
| *HMCN1 Ig37-Ig39* | ATTACCTGCACTGCTTCGGG | AGCATGGTTTAATTGGGTGGC |
| *HMCN1 Ig39-Ig41* | GATGGGCGTGCAATTGTGG | GTGCTTGTGCTGCTTGATCC |
| *HMCN1 VWA* | TGGGTCCTGTCATCACAATCTCC | AACTTTGCGTTGGTGCCTTTCC |
