# Supplementary material for: HMCN1 variants aggravate epidermolysis bullosa simplex phenotype
Source: J Exp Med. 2025 Feb 20;222(5):e20240827. doi: 10.1084/jem.20240827 (PMC11841684; doi:10.1084/jem.20240827)
Supplement: SourceData F2 — is the source file for Fig. 2. [file jem_20240827_sourcedataf2.pdf]

Ig27

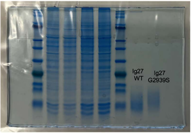

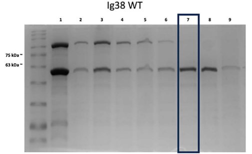

1– HMCN1-Ig38(WT) sup after nickel purification
2 – HMCN1-Ig38(WT) size exclusion elution Frac. 2
3 – HMCN1-Ig38(WT) size exclusion elution Frac. 3
4 – HMCN1-Ig38(WT) size exclusion elution Frac. 4
5 – HMCN1-Ig38(WT) size exclusion elution Frac. 5
6 – HMCN1-Ig38(WT) size exclusion elution Frac. 7
7 – HMCN1-Ig38(WT) size exclusion elution Frac. 8
8– HMCN1-Ig38(WT) size exclusion elution Frac. 9
9 – HMCN1-Ig38(WT) size exclusion elution Frac. 11
Lane 7 (boxed) was used in the main figure 2E

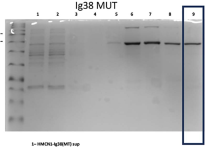

1– HMCN1-Ig38(MT) sup
2 – HMCN1-Ig38(MT) FW
3 – HMCN1-Ig38(MT) Wash 20mM Imidazole
4 – HMCN1-Ig38(MT) Wash 40mM Imidazole
5 – HMCN1-Ig38(MT) nickel elution Frac.3
6 – HMCN1-Ig38(MT) nickel elution Frac.4
7 – HMCN1-Ig38(MT) nickel elution Frac.5
8– HMCN1-Ig38(MT) size exclusion elution Frac. 6
9 – HMCN1-Ig38(MT) size exclusion elution Frac. 7
Lane 9 was used in the main figure 2E

Ig40 His4084Tyr

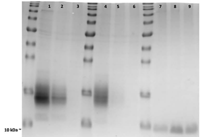

1 – HMCN1-Ig27(WT) Size Exclusion Elution Frac. 2
2 – HMCN1-Ig27(WT) Size Exclusion Elution Frac. 3
3 – Empty
4 – HMCN1-Ig27(MT) Size Exclusion Elution Frac. 2
5 – HMCN1-Ig27(MT) Size Exclusion Elution Frac. 3
6 – Empty
7 – HMCN1-Ig40(MT) Size Exclusion Elution Frac. 2
8 – HMCN1-Ig40(MT) Size Exclusion Elution Frac. 3 (was used in main figure 2E)
9 – HMCN1-Ig40(MT) Size Exclusion Elution Frac. 4

Ig40 WT

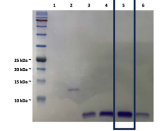

1 – HMCN1-Ig40(WT) Size Exclusion Elution Frac. 2
2 – HMCN1-Ig40(WT) Size Exclusion Elution Frac. 6
3 – HMCN1-Ig40(WT) Size Exclusion Elution Frac. 9
4 – HMCN1-Ig40(WT) Size Exclusion Elution Frac. 10
5 – HMCN1-Ig40(WT) Size Exclusion Elution Frac. 12
6 – HMCN1-Ig40(WT) Size Exclusion Elution Frac. 14

Lanes 3-6 purified Ig40
Lane 5 was used in the main Figure 2E
