# Supplementary figures and images for: HMCN1 variants aggravate epidermolysis bullosa simplex phenotype
Source: J Exp Med. 2025 Feb 20;222(5):e20240827. doi: 10.1084/jem.20240827 (PMC11841684; doi:10.1084/jem.20240827)

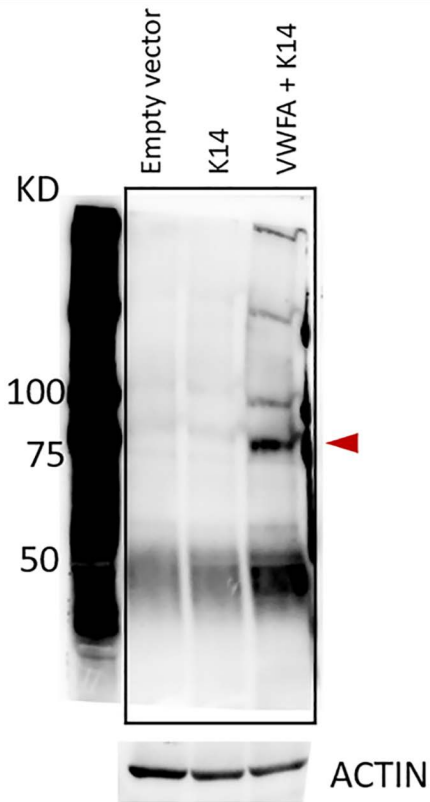

Supplement: SourceData F4 — is the source file for Fig. 4. [file jem_20240827_sourcedataf4.pdf]
